# Genetic Diversity of Indigenous Rice Varieties Cultivated by Mon-Khmer-Speaking Ethnic Communities in Thailand

**DOI:** 10.1186/s12284-025-00820-5

**Published:** 2025-07-04

**Authors:** Chayapa Sombat, Tanapon Seetaraso, Maneesawan Dansawan, Rattanasak Wongkomonched, Angkhana Inta, Siriphorn Jangsutthivorawat, Tonapha Pusadee, Jatupol Kampuansai

**Affiliations:** 1 Department of Biology, Faculty of Science, Chiang Mai University, Chiang Mai, 50202 Thailand; 2 Master Degree in Biology, Faculty of Science, Chiang Mai University, Chiang Mai, 50202 Thailand; 3 Department of Plant and Soil Sciences, Faculty of Agriculture, Chiang Mai University, Chiang Mai, 50202 Thailand

**Keywords:** *Oryza sativa*, Mon-Khmer, Genotyping by sequencing, Indigenous rice, Genome-wide, Northern Thailand

## Abstract

Rice (*Oryza sativa* L.) has been a vital staple crop in East and Southeast Asia for thousands of years, playing a key role in the development of human civilizations. Over time, different ethnic groups in these regions have selected rice varieties that suit their tastes and local growing conditions. As people migrated, they often brought their preferred rice varieties with them, contributing to the greater diversity of rice across regions. The Mon-Khmer-speaking peoples are believed to be the first settlers to introduce rice cultivation from southern China to northern Thailand during the Neolithic period. There are currently various indigenous rice varieties still being cultivated in Mon-Khmer communities of northern Thailand, but little is known about the genetic diversity of these rice varieties. This study examines 100 rice samples collected from 11 villages representing the Khmuic and Palaungic language branches of the Mon-Khmer group. Morphological analysis revealed differences in pericarp coloration, with Khmuic rice predominantly exhibiting off-white, brown, and black colors, while Palaungic rice tended to be lighter, including light and red shades. Genome-wide analysis identified two primary genetic clusters corresponding to these ethnolinguistic groups. Khmuic rice displayed high genetic homogeneity and characteristics of Subtropical Japonica rice, suggesting a stable lineage with limited seed exchange. In contrast, Palaungic rice exhibited greater genetic diversity, composed of both Japonica and Indica rice, likely due to extensive seed-sharing networks. Our findings provide insights into the relationship between ethnolinguistic groups and rice diversity, highlighting the importance of preserving indigenous rice varieties. This research also contributes to identifying novel genetic resources that may be useful for future rice breeding and improvement programs.

**Supplementary Information:**

The online version contains supplementary material available at 10.1186/s12284-025-00820-5.

## Introduction

Rice (*Oryza sativa* L.) is a crucial staple food crop that has been consumed by people in East and Southeast Asia for millennia, playing a significant role in human civilization's development over thousands of years (Sasaki 2001). Archaeobotanical evidence indicates that rice cultivation first occurred in the Yangtze River basin around 8,000 to 7,700 years B.P. (Fuller et al. 2009). Nonetheless, it is proposed that the domestication and cultivation of rice began independently in various locations across Asia (Glaszmann 1988; Cheng et al. 2003). The river basins stretching from Nepal to northern Vietnam are noted for their high rice diversity, with the greatest diversity found in Taiwan (Alam et al. 2021). Within mainland Southeast Asia, Yunnan Province in southern China is also recognized as a center of rice diversity, particularly for upland landraces, due to its complex geography and ecological heterogeneity (Gao et al. 2006).

Human selection and distribution have significantly contributed to rice diversity in different regions. Since rice is a staple food for most people in East and Southeast Asia, various ethnic groups have selected rice varieties that cater to their tastes and are well suited for local cultivation. Consequently, the types of rice varieties grown are often linked to specific ethnic groups, which also carry these selected varieties with them when they migrate to new areas, increasing the diversity of rice. While earlier models linked the spread of rice from southern China to Taiwan, the Philippines, and the Malay Archipelago to the migration of Austronesian-speaking populations around 2000–1000 years ago (Bellwood 1997), recent genomic studies suggest a more complex history. These studies indicate that Japonica rice likely dispersed southward from mainland Southeast Asia into the Malay Archipelago and later reached Taiwan via a south-to-north dispersal, facilitated by extensive trade and cultural exchange networks in the region (Gutaker et al. 2020; Alam et al. 2021).

Although Thailand is widely recognized as one of the world's leading rice-producing countries, the influence of population diversity on the distribution of local rice varieties remains largely unexplored. Archaeological and genetic evidence suggests that the people of Thailand share a close ancestral connection with populations in central and southern China, where *Oryza sativa* L. rice is believed to have been first domesticated. Archaeological evidence supports the southward movement of Neolithic populations from southern China to mainland Southeast Asia around 3,500 years ago. This migration is clearly reflected in the archaeological record of the Korat Plateau and surrounding regions in present-day Thailand (Higham 1996; 2015). This scenario is further supported by genome-wide analyses of ancient human remains, which suggest that Austroasiatic language speakers, particularly those from the Mon-Khmer subfamily, were the first to introduce rice cultivation technology from China to Thailand around 4,000–3,000 years ago, coinciding with the end of the Neolithic era (Lipson et al. 2018). Additional evidence from archaeobotanical data at Iron Age sites in northeast Thailand confirms the continued cultivation of domesticated rice during this period. Macro-remain findings from sites, such as Ban Non Wat and Non Ban Jak, highlight the central role of rice in agricultural subsistence amid changing climatic conditions in Thailand (Castillo et al. 2018).

Mon-Khmer speakers belong to the Austroasiatic language family, one of the five major linguistic families in Southeast Asia, alongside Sino-Tibetan, Tai-Kadai, Austronesian, and Hmong-Mien. They are believed to be descendants of prehistoric settlers in the region. Numerous scientific studies have consistently shown that Mon-Khmer-related ancestry has contributed to the genetic makeup of present-day Thai populations (Kutanan et al. 2021; Srithawong et al. 2022). Although most present-day Mon-Khmer individuals have been assimilated into other ethnic groups following the migration of Tai-Kadai-speaking peoples into Thailand around 2,000–1,000 years ago, some Mon-Khmer communities in Thailand, particularly in the north, have retained their distinct genetic makeup, cultural identity, and traditional knowledge of natural resource utilization (Kampuansai et al. 2023; 2024). This includes the preservation of native rice cultivation techniques and local rice varieties. In northern Thailand, Mon-Khmer speakers are classified into two primary linguistic branches: Khmuic and Palaungic (Schliesinger 2000). The Khmuic-speaking groups inhabit the Mekong River basin along the Thailand-Laos border and include the Khamu and Lua ethnic groups. The Palaungic branch consists of the Lwa, Dara-ang, and Lavue ethnic groups, who primarily reside in the western regions of northern Thailand along the Salween River at the Thailand-Myanmar border.

To explore the relationship between prehistoric Mon-Khmer-speaking populations and the distribution of local rice varieties in Thailand, we analyzed the genetic diversity and subpopulation structure of rice samples collected from Mon-Khmer-speaking communities, comparing those from the Khmuic and Palaungic branches. We hypothesize that certain indigenous rice varieties have been preserved within these communities through selective cultivation and intergenerational transmission, with their distribution influenced by ethnic differentiation. Using high-resolution genome-wide markers, our study establishes a comprehensive genetic framework for understanding the population structure of indigenous rice varieties shaped by human selection.

## Materials and Methods

### Plant Materials

**Fig. 1:**
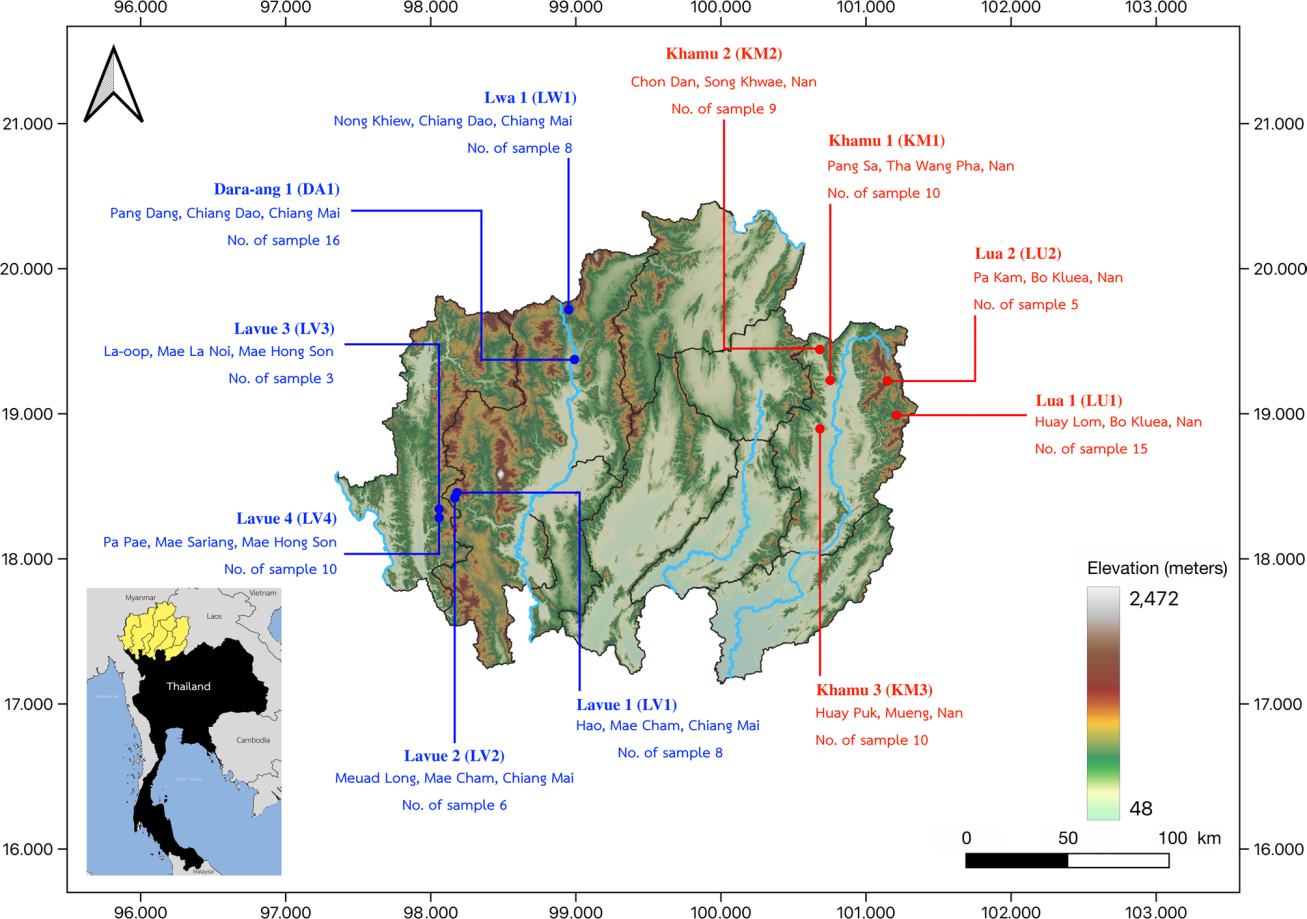
Map illustrating the geographical distribution of the 11 Mon-Khmer-speaking villages included in this study. Villages belonging to the Palaungic branch are represented by blue markers, while villages from the Khmuic branch are indicated by red markers. Background map was created using QGIS 3.6.0 (http://www.qgis.org/)

A total of 100 rice samples were collected from 11 Mon-Khmer communities, including five Khmuic villages in the eastern region (Nan Province) and six Palaungic villages in the western region (Chiang Mai and Mae Hong Son Provinces) of northern Thailand (Fig. 1). Each rice sample was assigned a sequential number: 1–51 for the Palaungic branch and 52–100 for the Khmuic branch (Supplementary Table 1). Information on variety names, planting methods, and usage was obtained through interviews. The locations, morphological characteristics, and photographs of the rice varieties were also documented.

### DNA Extraction, Genotyping, and Dataset Preparation

The collected rice seeds were cultivated in soil-filled pots and grown for one month before leaf samples were collected. Each leaf sample was ground in liquid nitrogen, and DNA was extracted using a DNeasy Plant Mini Kit (Qiagen, Hilden, Germany). DNA quantity and quality were assessed using a spectrophotometer. DNA samples that met quality criteria were sent for whole-genome SNP analysis using Genotyping by Sequencing (GBS) at Novogene, China.

Genomic data obtained through GBS underwent variant calling from the BAM file using BCFtools (Danecek et al. 2011), VCFtools (Danecek et al. 2011), Picard (Broad Institute 2019), and GATK (McKenna et al. 2010) to produce VCF files. These VCF files were then converted into BFILE format, comprising 62,226 SNPs, using PLINK v1.90b5.2 (Purcell et al. 2007) for use in population structure and relationship analyses. We also combined our newly genotyped data with the dataset with 2,966 accessions from the 3000 Rice Genomes Project (Wang et al. 2018) using the "bmerge" function in PLINK version 1.90b5.2. This resulted in a dataset comprising 3,066 samples representing rice varieties from 96 countries, with 62,226 SNP positions available for further analysis.

### Global Context of Mon-Khmer Rice and Subspecies Identification

To investigate the genetic background of our Mon-Khmer rice samples, we first analyzed the merged 3,066 rice dataset using principal component analysis (PCA), performed with the *smartpca* program from the EIGENSOFT package (Patterson et al. 2006). The resulting *eigenvectors* were visualized in R version 4.2.2 (R Core Team 2022) using the ggplot2 package. Genetic clustering and global relationships were then used to classify each rice variety into either the Japonica or Indica subspecies.

To infer population structure in a global context, ADMIXTURE v1.3.0 was utilized to investigate the genetic composition of Japonica and Indica rice dataset. Numerical genotype data were converted into .ped, .map, and .bed formats for analysis using ADMIXTURE software version 1.3.0 (Alexander et al. 2009), with the number of ancestral populations (K) ranging from 2 to 10. A total of 100 bootstrap iterations with varying random seeds were conducted, and the data visualization was performed using R software version 4.2.2 (R Core Team 2022).

### Pericarp Color and Seed Shape of Rice Grains

For each rice variety, 10 seeds were randomly selected and manually peeled. The pericarp color of each seed was visually examined and categorized based on a predefined color classification system. Six distinct pericarp color categories were identified: light (1), white (2), off-white (3), red (4), brown (5), and black (6) (Kiran et al. 2025). Additionally, the average length-to-width ratio of the 10 sampled rice grains was measured for each variety. These measurements were then categorized according to seed shape classification criteria into slender (> 3.00), medium (2.00–3.00), and bold (< 3.00) (Rice Department of Thailand 2023). Fisher’s exact tests were performed using R software version 4.2.2 (R Core Team 2022) to assess differences in Japonica and Indica rice varieties between the Khmuic and Palaungic groups based on seed morphological characterization.

### Diversity Indices and Population Structure Analyses

To dwell deeper into the genetic diversity within Mon-Khmer rice group, the newly generated genome-wide SNP dataset from 100 rice samples were converted to a genind object using the vcfR (Knaus and Grünwald 2017) and adegenet (Jombart 2008) packages in R version 4.2.2 (R Core Team 2022). Population assignments were specified based on linguistic group and rice type (Japonica or Indica), and diversity indices (including expected heterozygosity (He), observed heterozygosity (Ho), and effective number of alleles (Ne)) were calculated using the basic.stats function in the hierfstat package (Goudet 2005). Analyses were conducted at subgroup, village, and individual levels to evaluate within- and between-population diversity.

To assess population structure, we used ADMIXTURE v1.3.0 (Alexander et al. 2009) to analyze the genetic composition of our 100 Mon-Khmer rice samples, applying the same approach as for the global dataset. In brief, genotype data were first converted to PLINK format and examined across K values ranging from 2 to 10. We performed 100 bootstrap runs using different random seeds, and the optimal K value was determined based on the lowest cross-validation error and highest log-likelihood using a tenfold cross-validation procedure. The inferred ancestry proportions were then visualized as bar plots using R v4.2.2 (R Core Team 2022).

For genetic relationship analysis, pairwise genetic distances were computed using the EIGENSOFT *smartpca* tool (Patterson et al. 2006), and PCA plots were generated using ggplot2 in R. Pairwise F_ST_ values were then calculated separately for Japonica and Indica subspecies using genome-wide SNP data to assess genetic differentiation among Mon-Khmer-speaking villages. The F_ST_ matrices were generated using the–fst function in PLINK v1.90b5.2 (Purcell et al. 2007), based on predefined village groupings. Villages represented by fewer than two rice samples were excluded from the analysis to ensure meaningful population-level comparisons. The resulting values were visualized as clustered heatmaps using the *pheatmap* package in R (Kolde 2019), enabling the identification of genetically similar village clusters.

The maximum likelihood (ML) phylogenetic tree to infer deeper lineage-based relationships among samples was constructed using IQ-TREE v2.4.0 (Minh et al. 2020), applying the best-fit nucleotide substitution model (TVM + F + R2) selected by ModelFinder, with 1,000 ultrafast bootstrap replicates to evaluate the robustness of each branch. The tree was visualized in MEGA X v10.2.2 (Kumar et al. 2018).

Finally, Euclidean distance matrices were computed for each rice subspecies using the *dist( )* function in R, based on the same SNP dataset. These matrices were then used for Mantel and partial Mantel tests to assess correlations with language, geographic location, and elevation, using the *vegan* package in R (Oksanen et al. 2020). The input data included three variables: (1) language, categorized into two groups: Khmuic and Palaungic; (2) geographic coordinates, based on the latitude and longitude of each village; and (3) village elevation, measured in meters above sea level.

## Results

### Genetic Diversity and Relationships with Worldwide Rice Dataset

**Fig. 2:**
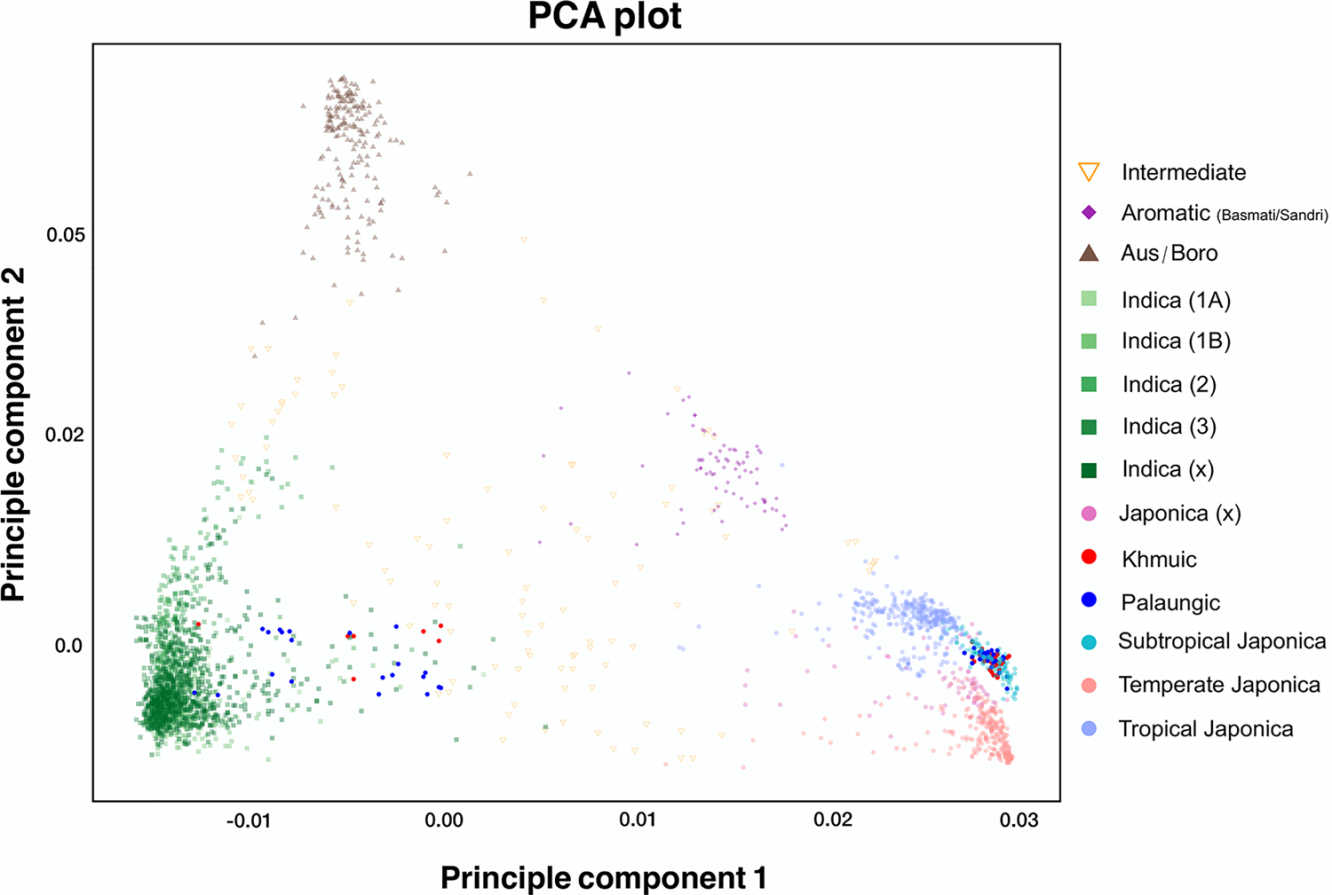
The PCA plot shows the genetic relationships among 3066 rice samples, with each point representing a distinct rice variety. The points are color-coded according to their group classifications based on rice types defined by the 3000 Rice Genomes Project, as indicated in the key on the right panel

We generated a genome-wide SNP dataset of 100 rice samples from the Palaungic and Khmuic language branches of the Mon-Khmer group and integrated them with global samples from the 3000 Rice Genomes Project. PCA analysis was then performed to determine the global context and identify subspecies (Fig. 2 and Supplementary Fig. 1). PCA based on rice types revealed that although Khmuic rice predominantly clustered within the Subtropical Japonica group, a few samples clearly belonged to the Indica cluster. In contrast, Palaungic samples exhibited more diverse patterns, incorporating contributions from Subtropical Japonica, and Indica lineages (Fig. 2). Based on the genetic clustering and global relationships shown in the PCA, our 100 Mon-Khmer rice samples can be classified into 72 Japonica and 28 Indica subspecies. The Khmuic rice includes 42 Japonica and 7 Indica samples, while the Palaungic group consists of 30 Japonica and 21 Indica varieties (Supplementary Table 1).

To further examine the genetic structure and ancestral composition of Mon-Khmer rice and global accessions, we applied ADMIXTURE version 1.3.0. The data were divided into two datasets: the Japonica dataset, which comprised 916 accessions, and the Indica dataset, which included 1,774 samples. A total of 376 accessions identified as “Intermediate”, “Aromatic (Basmati/Sandri)”, or “Aus/Boro” varieties by the 3000 Rice Genomes Project were excluded from the analysis.

In the Japonica group (Supplementary Fig. 2), most Mon-Khmer samples exhibited a high proportion of a single ancestry component, predominantly shared with Japonica accessions from Thailand, Laos, and Myanmar, with some presence also observed in accessions from Bhutan and India. Even as the number of ancestral populations (K) increased, Mon-Khmer Japonica rice remained dominated by a single ancestry, suggesting a conserved genetic lineage.

In contrast, the Indica group (Supplementary Fig. 3) revealed a more complex genetic structure in both Khmuic and Palaungic samples. Most individuals displayed multiple ancestry components, similar to those found in Indica accessions from South Asia (India and Bangladesh), Southeast Asia (Thailand, Laos, Myanmar, Cambodia, and Vietnam), and East Asia (China). Palaungic Indica samples, in particular, exhibited highly diverse ancestral profiles across individuals.

### Morphological Characteristics of Pericarp and Seed Shape

**Fig. 3:**
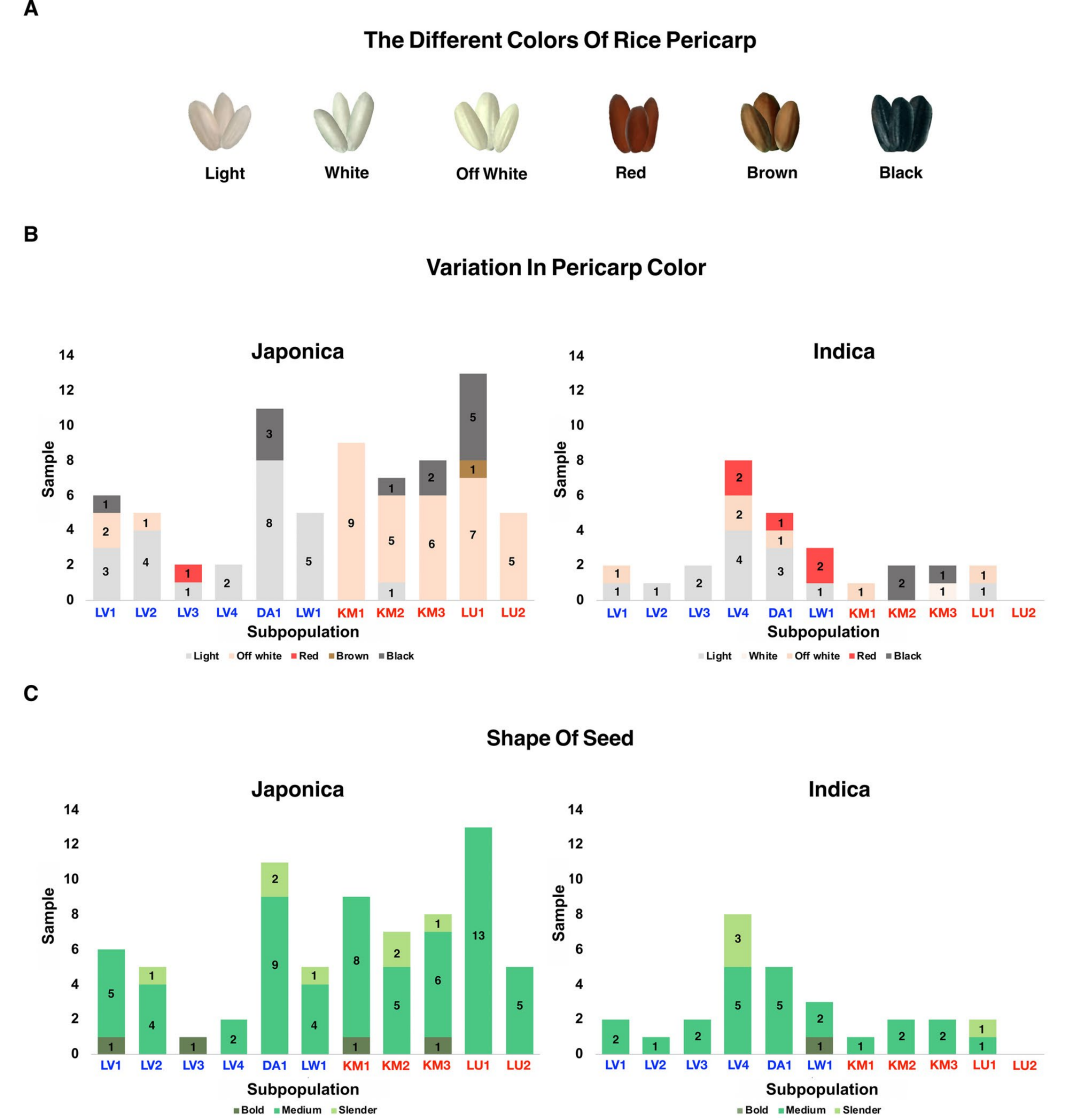
Rice pericarp color and seed shape of rice grown in Mon-Khmer-speaking villages of northern Thailand. Village names are labeled in blue and red to represent the Palaungic and Khmuic language branches, respectively. (A) Six categories of rice color variations which were observed in this study. (B) The number of rice samples categorized by pericarp color is shown separately for Japonica (left) and Indica (right) samples across villages. (C) Number of seed shapes in each village, displayed separately for Japonica (left) and Indica (right) rice samples

A total of 100 rice samples from Palaungic villages (LV1, LV2, LV3, LV4, DA1, and LW1) and Khmuic villages (KM, KM2, KM3, LU1, and LU2) were analyzed for variations in pericarp color and seed shape (Fig. 3). Note that one rice sample (No. 15 in Supplementary Table 1) exhibited two pericarp colors, possibly due to genetic heterogeneity or environmental influence. As a result, this sample was counted twice in the pericarp color analysis. Based on Fisher’s exact test, a statistically significant difference in pericarp color distribution was observed between rice from the Khmuic and Palaungic groups for both Japonica (p = 0.0001) and Indica (p = 0.0023) subspecies (Supplementary Table 2).

Among Japonica rice, Palaungic villages primarily cultivated light-colored grains (n = 23), particularly in DA1 (n = 8), whereas Khmuic villages exhibited a higher frequency of off-white (n = 32), brown, and black grains. Notably, red grains were absent in Khmuic Japonica rice but present in one Palaungic sample. For Indica rice, Palaungic samples generally displayed lighter pericarp colors (n = 12), while Khmuic samples showed a higher proportion of black grains (n = 3). Off-white grains were slightly more frequent in Palaungic (n = 4) than in Khmuic samples (n = 2) (Fig. 3B). These findings highlight ethnolinguistic differences in rice morphology, with Khmuic groups tending toward darker, pigmented grains and Palaungic groups favoring lighter pericarp colors across both Japonica and Indica varieties.

Regarding seed shape (Fig. 3C), both Japonica and Indica rice from the two ethnolinguistic groups exhibited similar trends, with medium grains being the most common. Slender and bold grain types were less frequent across all villages. Fisher’s exact tests (Supplementary Table 2) showed no statistically significant differences in seed shape distribution between the Khmuic and Palaungic groups within either Japonica (p = 0.6062) or Indica (p = 1.0000).

### Genetic Diversity and Population Structure of Mon-Khmer Rice

**Fig. 4:**
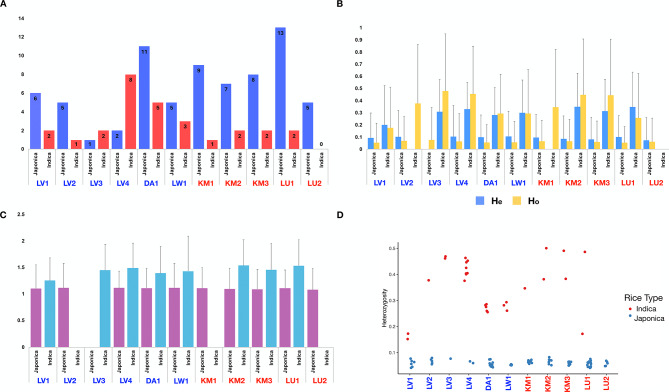
Genetic diversity of Mon-Khmer rice populations, separated by variety (Japonica and Indica) for each village. **A** Number of samples in each village; **B** observed heterozygosity (Ho) and expected heterozygosity (He); **C** effective allele number (Ne), a measure of allelic richness; **D** individual-level heterozygosity estimates for each rice sample

We evaluated genetic diversity in 100 rice samples collected from 11 Mon-Khmer-speaking villages using the number of rice subspecies per village, expected heterozygosity (He), observed heterozygosity (Ho), and effective allele number (Ne) (Fig. 4). Sample sizes varied by village and rice type, with LU1 and DA1 having the highest numbers of Japonica rice (n = 13 and 11, respectively), while Indica rice was more prevalent in LV4 and DA1. Among Indica samples, those from LV3 and LV4 displayed the highest observed heterozygosity values (up to ~ 0.48), whereas Japonica rice from LU2 and KM3 had low Ho values (0.05–0.07) and He values below 0.11. The effective allele number (Ne) was generally higher in Indica samples, with values exceeding 1.4 in several villages, including KM2 and LU1. Overall, Indica rice exhibited higher diversity than Japonica rice across all diversity indices.

These patterns were also reflected at the individual level, where Indica samples consistently showed greater heterozygosity than Japonica samples (Fig. 4D). Diversity statistics calculated at the language group level (Supplementary Table 3) further supported these observations: in both Khmuic and Palaungic groups, Indica rice demonstrated higher mean diversity values than Japonica rice. However, it is important to note that villages with small sample sizes, such as LV3 which had only one Japonica and two Indica samples, may limit the representativeness of diversity comparisons.

**Fig. 5:**
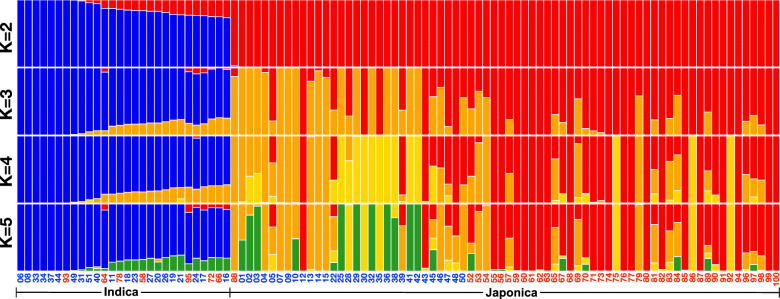
ADMIXTURE analysis of 100 rice samples for K values ranging from 2 to 5. Each rice variety is represented as a bar segmented into K distinct colors, indicating the estimated proportion of each genetic component. Different populations are separated by white lines. Village names are labeled in blue and red to represent the Palaungic and Khmuic language branches, respectively

The population structure of rice samples collected from Palaungic and Khmuic villages revealed that the lowest cross-validation error was observed at K = 2 (Supplementary Fig. 4), suggesting the presence of two major genetic components. At K = 2 (Fig. 5), two distinct genetic compositions (red and blue) were identified. The Khmuic populations exhibited relative genetic homogeneity, predominantly consisting of the red component, with only 7 out of 49 rice varieties showing the presence of the blue component. In contrast, the Palaungic populations displayed higher genetic diversity, comprising both red and blue components. As K increased from 3 to 10 (Supplementary Fig. 5), the greater genetic diversity within the Palaungic populations compared to the Khmuic populations became more pronounced.

**Fig. 6:**
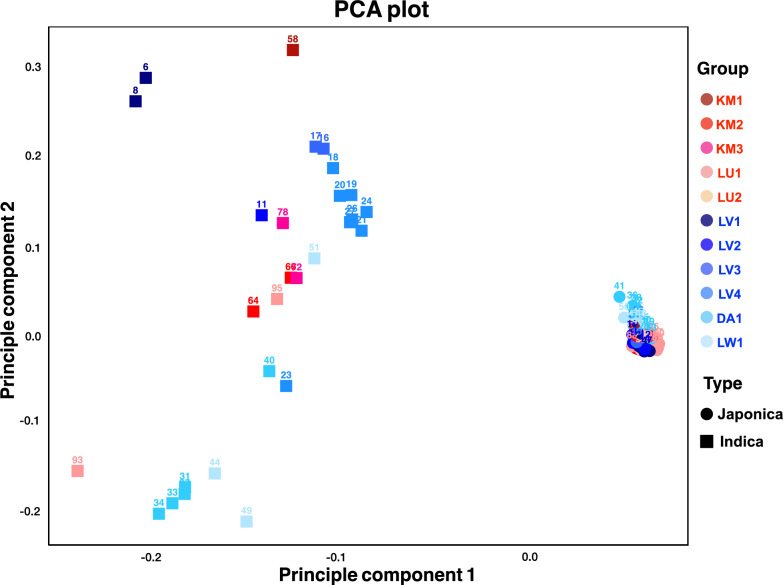
PCA analysis of 100 Mon-Khmer rice samples. Each individual is colored according to their village, as indicated in the key on the right panel. Samples from the Palaungic branch are shown in shades of blue, while those from the Khmuic branch are represented in shades of red

The genetic relationships among the Mon-Khmer rice samples were then examined using PCA analysis. The PCA plot strongly supports the ADMIXTURE results, indicating that rice associated with the Palaungic populations exhibits greater genetic diversity than that of the Khmuic group. As illustrated in Fig. 6, two distinct clusters are observed along PC1. Most Palaungic populations are widely dispersed on the left side of the plot, reflecting considerable within-group diversity, particularly evident in the DA1 and LV4 villages. In contrast, rice samples from the Khmuic group form a more compact cluster on the right side, suggesting a higher degree of genetic homogeneity.

To delve further into the genetic relationships at the population level, pairwise F_ST_ values were calculated separately for the Japonica and Indica subspecies (Supplementary Fig. 6 and 7). In the Japonica group, Khmuic villages (KM1–KM3, LU1) were genetically similar, while LU2 showed moderate differentiation but remained close to LU1. Palaungic villages LV1 and LV2 formed a tight cluster, while LV4 was more diverse. DA1 and LW1 showed similar genetic profiles, consistent with the PCA results. In the Indica group, Khmuic villages (KM2, KM3, LU1) exhibited low differentiation, indicating a weak population structure. Palaungic villages LV3 and LV4, as well as DA1 and LW1, also showed close relationships, suggesting shared ancestry or historical seed exchange. LV1 was notably distinct from all other villages.

### Distribution of Rice Lineages among Mon-Khmer Villages and Associated Factors

**Fig. 7:**
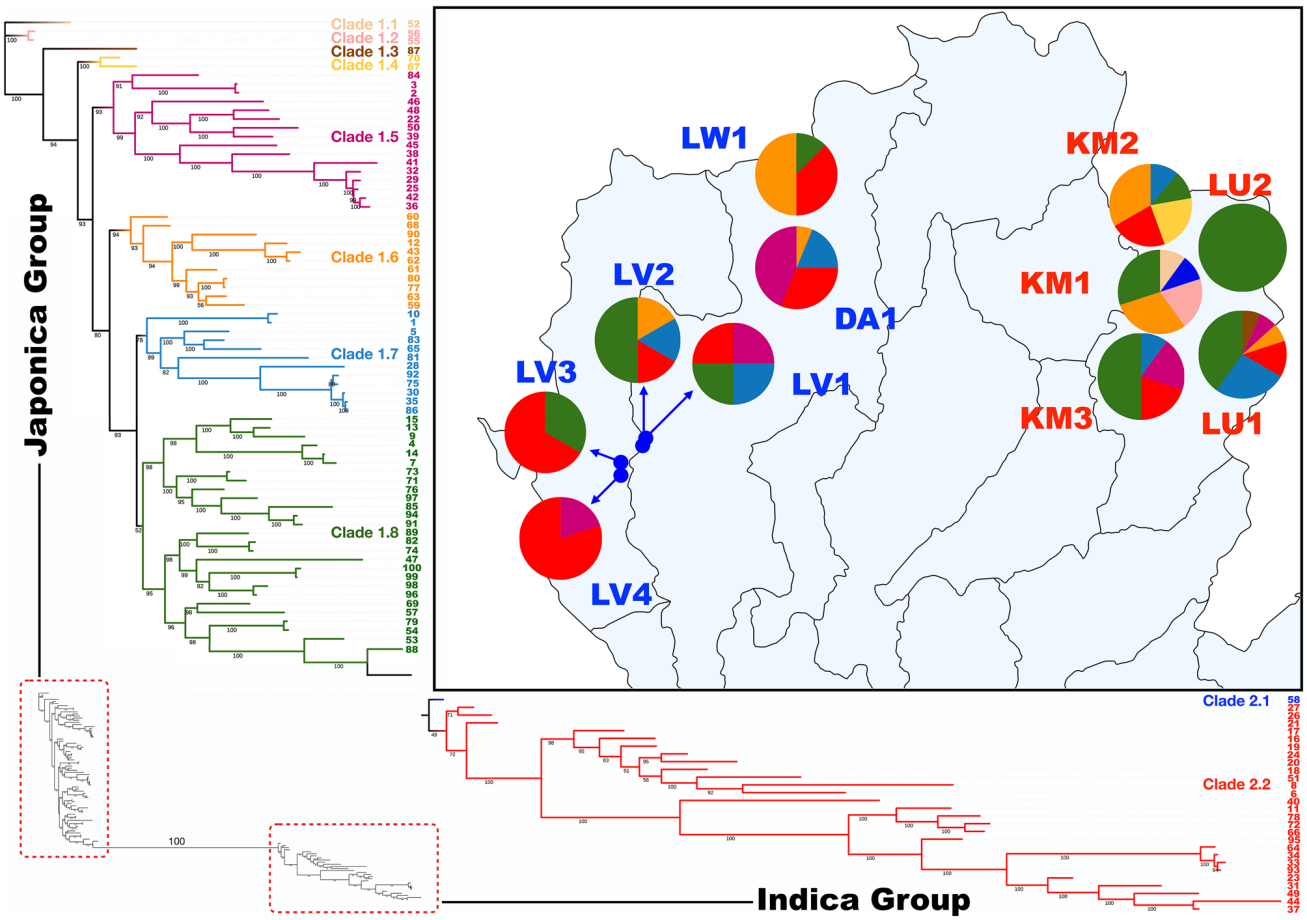
Geographic distribution and genetic relationships of rice populations across different villages. The right panel presents a map with pie charts depicting the genetic relationship of rice samples from each village, with colors corresponding to the clades in the maximum likelihood phylogenetic tree shown on the left. The background map was created using QGIS 3.6.0 (http://www.qgis.org/)

To describe the genetic relationships among our rice samples in greater lineage-based detail, we conducted a maximum likelihood (ML) phylogenetic analysis using IQ-TREE with the best-fit model (TVM + F + R2) and 1,000 ultrafast bootstrap replicates. The resulting tree revealed two clusters corresponding to the Japonica (clade 1) and Indica (clade 2) subspecies (Fig. 7, Supplementary Figs. 8 and 9). Within each clade, subclades were identified based on clustering patterns and bootstrap support values (bootstrap value ≥ 80), revealing genetic variation and relationships among rice varieties and villages. Rice varieties found within the same subclade suggested shared or closely related cultivar lineages, likely resulting from intra-community seed exchange or common selection criteria.

Among the Palaungic language branch, villages of the Lavue ethnic group (LV1–4) exhibited clear intra-ethnic differences. LV1 and LV2 predominantly consisted of Japonica rice belonging to clades 1.7 (light blue) and 1.8 (green), whereas LV3 and LV4 were primarily composed of Indica rice from clade 2.2 (red). Interestingly, rice from the Dara-ang (DA1) and Lwa (LW1) ethnic groups displayed greater within-village diversity, containing rice from multiple clades, with both Japonica and Indica lineages represented in relatively similar proportions.

Within the Khmuic-speaking villages, a more consistent color distribution pattern was observed in the Khamu ethnic group (KM1, KM2, and KM3), with dominant Japonica clades 1.6 (orange) and 1.8 (green), along with minor contributions from Indica clades 2.1 (blue) and 2.2 (red). Interestingly, certain Japonica rice lineages appeared to be specific to Khamu villages, including clades 1.1 (off-white), 1.2 (light pink), and 1.4 (yellow). Genetic differentiation was evident between the two Lua ethnic group villages (LU1 and LU2). LU1 displayed high genetic diversity, encompassing a wide range of clades, while LU2 contained only one rice clades, with clade 1.8 (green) as the predominant genetic component. Notably, a unique lineage, clade 1.3 (brown), was exclusively found in LU1 and absent from all other Khmuic-speaking villages. Although most Mon-Khmer rice lineages appear to differ between Palaungic- and Khmuic-speaking villages, certain lineages, such as clades 1.5 (pinkish purple), 1.6 (orange), 1.7 (light blue) and 1.8 (green), were shared between these two language groups, potentially reflecting ancestral connections or historical interactions (Fig. 7).

To investigate the correlation between genetic differentiation and related factors, including language, geographic location, and elevation, we performed Mantel and partial Mantel tests (Supplementary Table 4). For the Japonica group, both linguistic and geographic distances showed significant associations with genetic distance when assessed individually (r = 0.1265, p = 0.001 and r = 0.0915, p = 0.036, respectively), whereas elevation had no significant effect (r =–0.0920, p = 0.937). The effect of language remained significant after controlling for either geography (r = 0.0989, p = 0.034) or elevation (r = 0.1551, p = 0.001), indicating a robust relationship between linguistic affiliation and genetic differentiation. In contrast, geography was only significant when controlling for elevation (r = 0.1407, p = 0.001), but not when controlling for language (r = -0.0459, p = 0.731), suggesting partial overlap between language and geographical factors. Elevation did not exhibit a significant effect in any test. Overall, these results highlight the consistent and independent role of language in shaping the genetic structure of Japonica rice, alongside geographical location.

In contrast, for the Indica group, elevation showed the strongest association with genetic distance, both in the Mantel test (r = 0.2449, p = 0.016) and in partial Mantel tests, remaining significant when controlling for either language (r = 0.2490, p = 0.026) or geography (r = 0.2192, p = 0.046). Geographic distance was only significant when language was controlled (r = 0.1344, p = 0.003), but not when controlling for elevation (r = -0.0088, p = 0.515), suggesting that elevation may account for much of the spatial structure. Language did not show a significant effect in any test. These findings indicate that elevation is the dominant factor influencing the genetic structure of Indica rice, while linguistic background has a limited impact.

## Discussion

### Origins and Historical Context of Mon-Khmer Rice

Based on the genome-wide SNP dataset comprising 3,066 rice varieties, including samples newly generated in this study and global accessions from the 3000 Rice Genomes Project, the PCA plots (Fig. 2) and phylogenetic trees (Supplementary Figs. 8 and 9) reveal a distinct separation between Japonica and Indica clusters within the Mon-Khmer rice samples. These genetic patterns suggest the persistence of reproductive or sociocultural barriers that limit gene flow between Japonica and Indica rice types in local farming contexts. This observation is consistent with previous research on the origin and differentiation of aromatic rice varieties, which found that despite geographic proximity and overlapping cultivation areas, gene flow between Indica and Japonica remains limited (Civáň et al. 2015a). Reproductive isolation, together with farmers’ preferences for specific traditional varieties, likely contributes to the continued genetic distinctiveness between these two major rice subspecies.

We found that rice populations cultivated by Mon-Khmer ethnolinguistic groups predominantly align with Subtropical Japonica rice varieties, particularly in Khmuic-speaking communities. These Japonica rice types, well-suited to mid-altitude and highland environments, have been grown in the mountainous regions of Southeast Asia for centuries (Courtois et al. 2013). The primary center of Japonica rice domestication is identified in China, particularly the Yangtze River Valley, around 6,000 B.C. (Larson et al. 2014). This area is also suggested to be the ancestral homeland of the Mon-Khmer-speaking populations, as supported by genetic data (Lipson et al. 2018). The link between the Japonica rice variety and the human ancestry of the communities in our study further supports that the spread of this rice variety and cultivation techniques from China to Thailand was facilitated by the southward migration of Austroasiatic-speaking peoples into mainland Southeast Asia (Castillo et al. 2016; Fuller et al. 2009).

The Khmuic-speaking people had been found to be the pre-historic descendances of ancient communities in mainland Southeast Asia. The human genomic data of these people exhibited the remnants of the 2,378–3,071-year-olds ancient DNA discovered at Tam Pa Ling and Tham Hang Caves in Laos (Kampuansai et al. 2023). The prominent presence of homogenized Subtropical Japonica traits within the Khmuic rice populations (Fig. 2 and 5) supports the hypothesis that the ancestors of Khmuic-speaking groups participated in early rice farmers of Southeast Asia. Linguistic and archaeological studies substantiate this scenario, suggesting that Khmuic-speaking populations were among the early adopters of rice agriculture in Southeast Asia, significantly contributing to the dissemination of Japonica varieties adapted to high-altitude conditions (Varinrak 2011). This historical context may clarify the strong genetic conservation and preserved across generations observed in Khmuic rice, reflecting an ancient and sustained lineage cultivated under relatively isolated and stable agricultural systems. In contrast, Palaungic rice populations exhibit a more complex genetic profile, characterized by greater diversity and the presence of both Japonica and Indica lineages (Fig. 4). When comparing the two major subspecies, Indica rice shows higher genetic variation than Japonica at both the individual and village levels (Figs. 4 and 6), suggesting that Indica varieties may have experienced broader seed exchange or adaptation to diverse environments. The substantial presence of Indica rice in Palaungic speaking communities point to significant historical interactions between Palaungic rice cultivators and communities growing Indica rice, which originated in the Indian subcontinent and spread into Southeast Asia through ancient trade, migration, and cultural exchanges (Castillo 2011). Archaeological and genetic evidence have shown extensive agricultural exchanges along historical trade routes linking mainland Southeast Asia with the larger Indian subcontinent's inland and maritime trade networks. These exchanges facilitated the movement of human lineages and diverse agricultural resources, including Indica rice varieties (Castillo et al. 2016; Kampuansai et al. 2024). Consequently, the increased genetic diversity within Palaungic rice populations likely reflects a dynamic agricultural history shaped by seed exchange among different ethnolinguistic groups. The high degree of variation and mixed ancestral profiles observed in present-day Palaungic Indica rice populations underscores their adaptation to diverse ecological and cultural settings. Interestingly, elevation was found to correlate with genetic distance only in Indica varieties, not in Japonica (Supplementary Table 4). This supports the idea that the high genetic diversity and multiple ancestral components observed in Indica rice may reflect its broader adaptation to varying environmental conditions across different elevations in the highlands of northern Thailand. This genetic diversity may have contributed to improved local adaptation, yield stability, and resilience to stress in the culturally diverse upland farming systems (Civáň et al. 2015b; Gutaker et al. 2020).

### Comparative Diversity Between Rice of Palaungic and Khmuic-speaking Populations

Pericarp color and seed shape are key morphological characteristics of rice that exhibit variation across ethnic groups. Human selection for desirable taste profiles has significantly shaped the genetic traits of cultivated rice, with genes influencing starch texture and grain softness, such as those regulating amylose content, being strongly selected to align with the dietary preferences of different ethnic communities (Sweeney and McCouch 2007). In our analysis of rice morphology among Mon-Khmer-speaking communities in Thailand, we observed that rice varieties from the Palaungic language branch exhibit greater diversity in seed morphology compared to those from the Khmuic branch. While medium-grain rice is the predominant type in both groups, Palaungic rice populations are characterized by lighter pericarp colors, whereas Khmuic rice populations display darker grains, primarily off-white, black, and brown (Fig. 3). These findings suggest that variations in rice color may reflect selection pressures driven by the taste preferences and cultivation practices of Mon-Khmer farmers. Historically, early farmers favored non-shattering rice varieties to reduce seed dispersal, facilitating easier harvesting and increased yields. This selection pressure contributed to the enlargement of grain size, leading to cultivated rice being larger than its wild relatives. Additionally, human-mediated selection has played a fundamental role in developing rice varieties adapted to diverse environmental conditions, thereby enhancing the extensive genetic diversity observed in present-day rice populations (Fuller et al. 2009).

Our genome-wide analysis supports the morphological findings by revealing different genetic backgrounds between Khmuic and Palaungic rice populations. ADMIXTURE and PCA analyses (Figs. 5 and 6) indicate that Palaungic rice exhibits greater genetic diversity and multiple ancestral components, likely reflecting historical interactions and possible seed exchange with external rice-growing communities, as discussed earlier. Within the Palaungic branch, we observed varying preferences for rice types within the Lavue ethnic group. While LV1 and LV2 predominantly cultivate Japonica varieties, LV3 and LV4 primarily grow Indica rice (Fig. 3 and 4). Notably, ethnographic information indicates that villagers from LV1 and LV2 share extended family lineages, as do those from LV3 and LV4 (Prachuabmoh et al. 2012), suggesting that rice variety preferences may be shaped by seed exchange practices among closely related kin groups. Furthermore, the geographic context reveals that LV3 and LV4 are situated along accessible road networks and maintain frequent interactions with external communities, potentially facilitating the introduction and adoption of Indica rice varieties, which are commonly cultivated by lowland populations in northern Thailand. These intra-ethnic differences in rice cultivation therefore appear to be influenced not solely by ethnolinguistic affiliation, but also by kinship structures, geographic accessibility, and the degree of external contact. All of which play critical roles in shaping human-mediated seed selection and agricultural decisions.

Rice varieties cultivated by the Palaungic-speaking Dara-ang (DA1) and Lwa (LW1) groups exhibit a widely scattered distribution across the phylogenetic tree, likely indicating significant population admixture resulting from historical interactions with multiple communities (Fig. 7 and Supplementary Fig. 9). The Dara-ang and Lwa communities in Thailand are known to have migrated from neighboring regions within the past few centuries. Given the small population sizes involved in these migrations, it is unlikely that they carried their original rice varieties due to limited agricultural labor. Upon establishing permanent settlements in Thailand, rice cultivation became central to their subsistence; however, their rice varieties appear to have been primarily introduced from surrounding ethnic groups. Noteworthy, a prior study on human DNA from Palaungic-speaking communities identified genetic admixture with several ethnolinguistic groups, particularly the Sino-Tibetan-speaking Karen people, likely due to interethnic marriages (Kampuansai et al. 2024). This raises the possibility that rice varieties from Karen communities, have been introduced and subsequently cultivated within Palaungic-speaking villages. The exclusive presence of rice genetic lineages corresponding to 2.2 (red) (Fig. 7) within Palaungic-speaking groups may thus be a result of historical exchanges with Karen agricultural traditions. Unfortunately, genomic data for Karenic rice is not available in the 3000 Rice Genomes Project or other public genomic databases. As a result, it is not possible to clearly assess shared ancestry or seed exchange between Karenic and Palaungic rice populations. A broader ethnically based study on rice varieties will be essential for reconstructing the evolutionary history of upland rice in Southeast Asia.

In contrast, rice populations cultivated by Khmuic-speaking communities exhibit greater genetic homogeneity, indicative of restricted gene flow and relative isolation from other groups. This pattern is likely shaped by traditional agricultural practices that emphasize lineage preservation within communities (Molina et al. 2011). Within the Khmuic community, a high degree of genetic similarity is observed between rice varieties cultivated by the Khamu (KM1-3) and Lua (LU1-2) ethnic groups, as evidenced by the similar proportions of genetic components and their tight clustering patterns (Figs. 6 and 7). This genetic similarity aligns with the previously reported close shared ancestry of these groups, as inferred from genome-wide human genetic markers (Kampuansai et al. 2023). The strong relatedness among Khmuic-speaking populations may have facilitated the exchange of agricultural knowledge, including rice cultivation practices, thereby contributing to the sharing of rice varieties. Interestingly, rice cultivated by the Lua ethnic group from two villages exhibits distinct genetic differentiation. While the LU1 population cultivates a diverse range of rice varieties, LU2 cultivates only a limited number of varieties and displays a unique genetic composition. This differentiation may be influenced by varying degrees of interaction with external communities. Notably, the LU1 village is located near the Royal Initiative Station of H.R.H. Princess Maha Chakri Sirindhorn, where local Lua rice varieties are actively supported for preservation and cultivation. In contrast, the LU2 village is situated in a remote mountainous region, where reduced external interaction likely limits opportunities for rice seed exchange.

Although the overall genetic profiles of rice cultivated by Palaungic and Khmuic communities are largely distinct, some rice lineages are found in both linguistic branches (Fig. 7). These findings suggest two possible explanations. Our first hypothesis proposes that the selection of rice varieties may have occurred within a common ancestral population prior to the divergence of the two linguistic branches. Genetic evidence indicates that all Mon-Khmer-speaking populations of Mainland Southeast Asia share a common ancestry, tracing back to a population in Central China that introduced rice cultivation technology to Southeast Asia by the end of the Neolithic period (Lipson et al. 2018). It is possible that the selection of cultivated rice varieties took place during this migration and was subsequently preserved within certain Mon-Khmer-speaking communities. Our second hypothesis suggests that the presence of shared rice varieties results from historical seed exchange between Palaungic and Khmuic communities through trade networks. Despite residing in different regions of northern Thailand, these groups were connected via inland commercial routes, historically known as “Northern Caravan Trade Routh” or “Ma-Tang, Wua-Tang” (a Thai term meaning “carrying on horses, carrying on cows”) (Chusit 2002). This trade network linked villages and cities from southwest China through northern Thailand to southern Myanmar, facilitating interactions that may have enabled the exchange of rice seeds. Our analyses also supported the partial correlation between geographic location and genetic differentiation in Mon-Khmer rice populations (Supplementary Table 4). Villages such as the Palaungic-speaking DA1 and LW1 are situated along more accessible routes, which may have facilitated interactions with both eastern and western communities. This geographic positioning likely enabled the introduction of diverse rice varieties through various channels. In contrast, villages like Khmuic-speaking LU and KM, located in more remote and isolated areas, likely had limited external contact, resulting in more genetically uniform rice populations. Although, historical trade and seed exchange have been shown to influence the distribution of upland rice populations across Southeast Asia, only a limited number of rice varieties can successfully establish in different regions and communities. Their survival depends on their adaptive capacity to local environmental conditions (Gutaker et al. 2020).

### Implications for Rice Conservation and Future Research

Our findings highlight the crucial role of ethnolinguistic identity in shaping rice genetic diversity. The greater genetic diversity seen in Palaungic rice populations showcases a dynamic seed exchange system that has historically promoted genetic mixing. In contrast, the genetic uniformity found in Khmuic rice populations indicates traditional farming practices that prioritize lineage conservation and the preservation of ancestral varieties (Kulsawakul et al. 2019). Such genetically preserved rice populations are vital reservoirs of genetic diversity, valuable for breeding programs to improve stress tolerance and climate resilience in contemporary rice varieties.

Future research efforts should prioritize genome-wide association studies (GWAS) to uncover genomic regions controlling critical agronomic traits, such as pericarp pigmentation, grain shape, and traits related to adaptation to various environments. Such studies would provide a deeper understanding of the molecular basis underlying phenotypic variation in traditional rice cultivars (Wang et al. 2017). Moreover, exploring historical seed exchange networks among ethnolinguistic groups will shed further light on the cultural practices and social interactions that have contributed significantly to rice populations' current genetic structure and diversity (Castillo et al. 2016; Mbanjo et al. 2020). Broadening research to include additional ethnolinguistic communities across various ecological niches would significantly advance our understanding of how cultural practices, environmental adaptation, and human-mediated selection collectively influence rice genetic evolution and diversity (Stein et al. 2018).

## Conclusion

Our comprehensive morphological and genetic analyses of rice populations cultivated by the Khmuic and Palaungic ethnolinguistic groups of the Mon-Khmer subfamily reveals notable ethnic-based differences in pericarp color, genetic structure, and phylogenetic classification. Overall, the rice populations of Khmuic-speaking communities show strong genetic conservation of Subtropical Japonica rice, likely due to limited gene flow, localized selection pressures, and few opportunities for seed exchange. In contrast, Palaungic rice populations exhibit greater diversity in both Japonica and Indica rice, probably because of their higher level of interaction with outside communities. Our findings offer valuable insights into the historical development of rice agriculture and how ethnolinguistic groups have influenced rice genetic diversity over time. Understanding these patterns is important for future rice breeding programs, conservation efforts, and sustainable agricultural practices in Southeast Asia.

## Supplementary Information

Supplementary Material 1.

Supplementary Material 2.

## Data Availability

Rice genomic datasets generated and analyzed during the current study are available in the Zenodo repository (https://doi.org/10.5281/zenodo.15093208).
