# Supplementary material for: Genetic Diversity of Indigenous Rice Varieties Cultivated by Mon-Khmer-Speaking Ethnic Communities in Thailand
Source: Rice (N Y). 2025 Jul 4;18:60. doi: 10.1186/s12284-025-00820-5 (PMC12227406; doi:10.1186/s12284-025-00820-5)
Supplement: Supplementary file 1 — Supplementary Material 1. [file 12284_2025_820_MOESM1_ESM.docx]

**Supplementary Figure 1.** PCA plot illustrating the genetic relationships among worldwide rice populations. Each spot represents a rice variety, with different colors and shapes corresponding to countries, as indicated in the right panel.

**Supplementary Figure 2A.** The ancestry components of Japonica rice varieties, based on 916 samples analyzed in this study and from the 3K Rice Genome Project, are shown for K values ranging from 2 to 10. Population structure was inferred using ADMIXTURE version 1.3.0. The barplots represent individuals sorted by population, including samples cultivated by Mon-Khmer ethnolinguistic groups in regions from Thailand to India.

**Supplementary Figure 2B.** The ancestry components of the remaining Japonica rice varieties after India, including those from East Asia, Africa, Europe, and the Americas, are shown for K values ranging from 2 to 10. The plots illustrate the population structure estimated by ADMIXTURE version 1.3.0, with individuals grouped by country.

**Supplementary Figure 3A.** The ancestry components of 1,774 Indica rice varieties, based on samples analyzed in this study and from the 3K Rice Genome Project, are shown for K values ranging from 2 to 10. Population structure was inferred using ADMIXTURE version 1.3.0. The barplots represent individuals sorted by population, including samples cultivated by Mon-Khmer ethnolinguistic groups in regions from Thailand to India.

**Supplementary Figure 3B.** The ancestry components of the remaining Indica rice varieties after India, including those from East Asia, Africa, Europe, and the Americas, are shown for K values ranging from 2 to 10. The plots illustrate the population structure estimated by ADMIXTURE version 1.3.0, with individuals grouped by country.

**Supplementary Figure 4.** The cross-validation results from the analysis of 100 Mon-Khmer rice samples using ADMIXTURE version 1.3.0 are shown for K values ranging from 2 to 10

**Supplementary Figure** **5**. ADMIXTURE analysis of 100 rice samples for K values ranging from 2 to 10. Each rice variety is represented as a bar segmented into K distinct colors, indicating the estimated proportion of each genetic component. Different populations are separated by white lines. Sample names are labeled in blue and red to represent the Palaungic and Khmuic language branches, respectively.

**Supplementary Figure 6.** Heatmap of pairwise F_ST_ values among Japonica rice samples from Mon-Khmer-speaking villages. The matrix represents genetic distances between village-level populations, with Khmuic and Palaungic groups distinguished by differently colored labels on their village codes.

**Supplementary Figure 7**. Heatmap of pairwise F_ST_ values among Japonica rice samples from Mon-Khmer-speaking villages. The matrix represents genetic distances between village-level populations, with Khmuic and Palaungic groups distinguished by differently colored labels on their village codes.

**Supplementary Figure 8.** A ML phylogenetic tree illustrating the genetic relationships among rice populations cultivated by different language communities. The terminal nodes are colored according to language branch: Palaungic (blue) and Khmuic (red).

**Supplementary Figure 9.** A ML phylogenetic tree illustrating the genetic relationships among rice populations cultivated by different ethnolinguistic groups. The terminal nodes are colored according to different ethnic groups: Khamu (blue), Lua (purple), Lwa (yellow), Lavue (orange), and Dara-ang (pink).
