# Supplementary material for: Genetic Diversity of Indigenous Rice Varieties Cultivated by Mon-Khmer-Speaking Ethnic Communities in Thailand
Source: Rice (N Y). 2025 Jul 4;18:60. doi: 10.1186/s12284-025-00820-5 (PMC12227406; doi:10.1186/s12284-025-00820-5)
Supplement: Supplementary file 2 — Supplementary Material 2. [file 12284_2025_820_MOESM2_ESM.docx]

| **Supplementary Table 1. List of rice seeds collected from Mon-Khmer ethnic communities in northern Thailand. Rice no. 1-51 are from the Palaungic branch and no. 52-100 are from Khmuic branch.** | | | | | | | | | | | | | | |  |
| --- | --- | --- | --- | --- | --- | --- | --- | --- | --- | --- | --- | --- | --- | --- | --- |
| **Number** | **Village Code** | **Name Code** | **Rice Name**  **(in Thai)** | **Rice name**  **(in English)** | **Pericarp color** | **Average seed length (cm)** | **Average seed width (cm)** | **Average length/width** | **Seed shape** | **Type** | **N_Loci** | **N_Het** | **Heterozygosity** | **Picture of seed** | |
| 1 | LV1 | RLW1V01 | พันธุ์ขาว (บือสุคี) | Phan Khao (Bue Suki) | Light | 6.156 | 3.348 | 1.888 | Bold | japonica | 28930 | 2221 | 0.0768 | 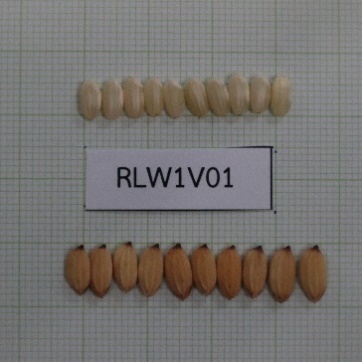 | |
| 2 | LV1 | RLW1V08 | เปลือกแดง | Plook Daeng | Light | 6.229 | 2.894 | 2.203 | Medium | japonica | 31501 | 2001 | 0.0635 | 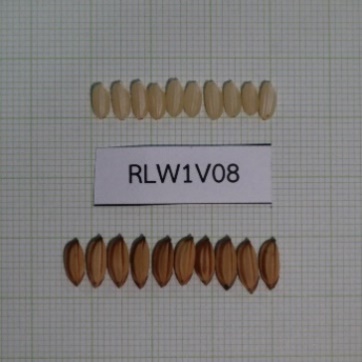 | |
| 3 | LV1 | RLW1V10 | เปลือกแดง | Plook Daeng | Light | 6.467 | 2.811 | 2.237 | Medium | japonica | 28735 | 1618 | 0.0563 | 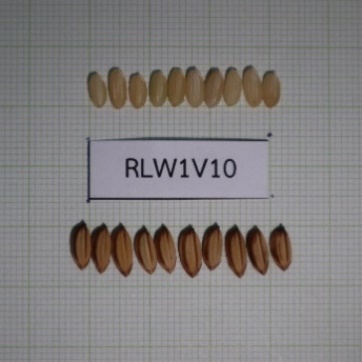 | |
| 4 | LV1 | RLW1V12 | เปลือกแดง | Plook Daeng | Off-white | 7.225 | 2.481 | 2.798 | Medium | japonica | 33760 | 1523 | 0.0451 | 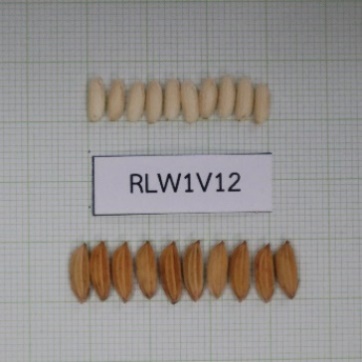 | |
| 5 | LV1 | RLW1V13 | เหนียวดำ | Niao Dam | Black | 7.202 | 2.975 | 2.415 | Medium | japonica | 28002 | 1886 | 0.0674 | 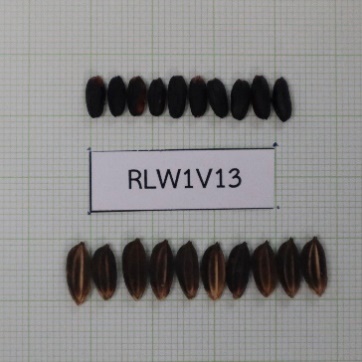 | |
| 6 | LV1 | RLW1V15 | บือข่า (เจ้าดอย) | Bue Kha (Chao Doi) | Off-white | 6.561 | 3.140 | 2.063 | Medium | indica | 54796 | 9454 | 0.1725 | 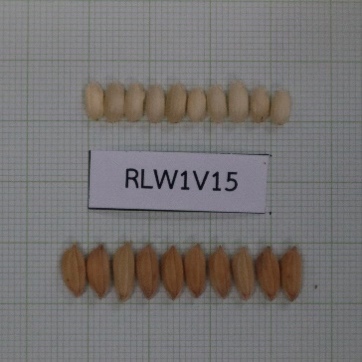 | |
| 7 | LV1 | RLW1V16 | เหนียวใหญ่ | Niao Yai | Off-white | 6.956 | 3.123 | 2.228 | Medium | japonica | 56731 | 2340 | 0.0412 | 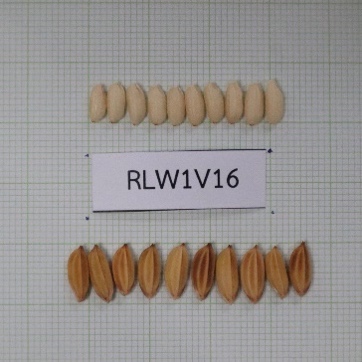 | |
| 8 | LV1 | RLW1V17 | มะลิลัวะ | Mali Lua | Light | 7.329 | 2.720 | 2.873 | Medium | indica | 54773 | 8306 | 0.1516 | 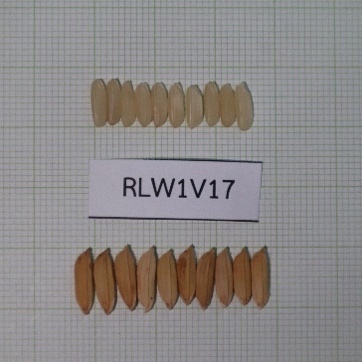 | |
| 9 | LV1 | RLW2V01 | เงาะกะเซ | Ngo Ka Se | Light | 6.992 | 2.717 | 2.507 | Medium | japonica | 34170 | 2031 | 0.0594 | 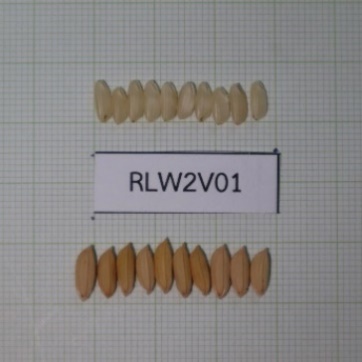 | |
| 10 | LV1 | RLW2V02 | พรือซากรัก | Prue Sak Rak | Light | 6.780 | 3.229 | 2.106 | Medium | japonica | 31447 | 2110 | 0.0671 | 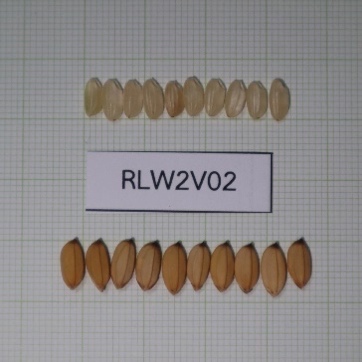 | |
| 11 | LV2 | RLW2V04 | เปลือกแดง | Plook Daeng | Light | 6.589 | 3.115 | 2.081 | Medium | indica | 31808 | 12003 | 0.3774 | 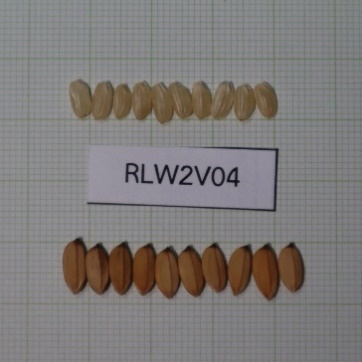 | |
| 12 | LV2 | RLW2V06 | มะลิดอย | Mali Doi | Light | 8.399 | 2.612 | 3.265 | Slender | japonica | 33403 | 1838 | 0.055 | 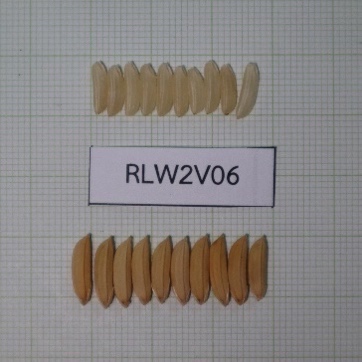 | |
| 13 | LV2 | RLW2V07 | ฮ๊อปเชือน | Hop Chuean | Light | 5.873 | 2.501 | 2.360 | Medium | japonica | 28022 | 2223 | 0.0793 | 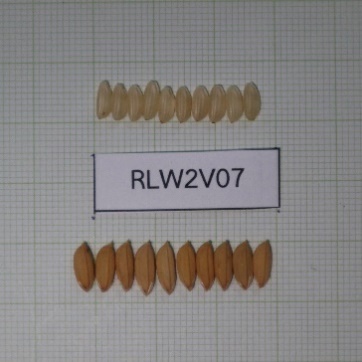 | |
| 14 | LV2 | RLW2V09 | เหนียวดอย (เงาะปิ๊บ) | Niao Doi (Ngo Pip) | Off-white | 7.344 | 2.561 | 2.889 | Medium | japonica | 30097 | 2205 | 0.0733 | 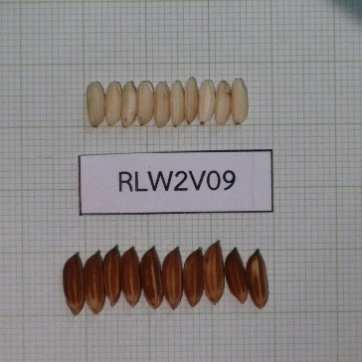 | |
| 15 | LV3 | RLW3V01 | ข้าวไร่ (เหงาะมา) | Khao Rai (Ngo Ma) | Red and  Light | 5.887 | 2.937 | 1.958 | Bold | japonica | 26711 | 2046 | 0.0766 | 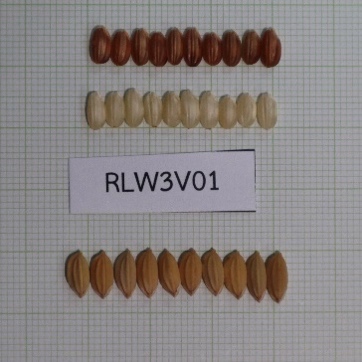 | |
| 16 | LV3 | RLW3V02 | ข้าวนา ข้าวเหลืองกลาง (เหงาะฉิก) | Khao Na Khao Luang Klang (Ngo Chik) | Light | 7.270 | 3.057 | 2.402 | Medium | indica | 27586 | 12936 | 0.4689 | 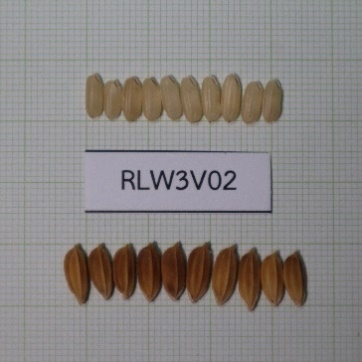 | |
| 17 | LV3 | RLW3V03 | ข้าวนา เหลืองใหญ่ | Khao Na Luang Yai | Light | 7.546 | 2.739 | 2.747 | Medium | indica | 30193 | 13927 | 0.4613 | 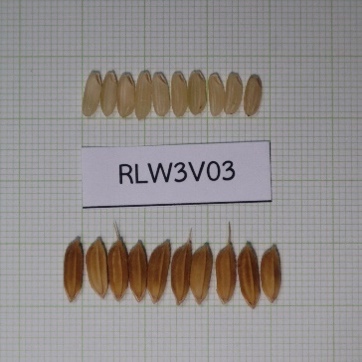 | |
| 18 | LV4 | RLW7V01 | เฮาะฉึมฉึม (นา) | Hao Chim Chim (Na) | Light | 6.594 | 2.918 | 2.277 | Medium | indica | 19417 | 7290 | 0.3754 | 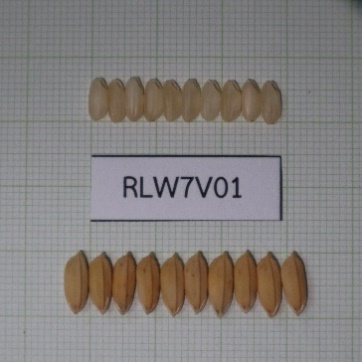 | |
| 19 | LV4 | RLW7V02 | เฮาะพะยุย (ไร่) | Hao Pa Yui (Rai) | Light | 7.901 | 2.834 | 2.846 | Medium | indica | 21195 | 9587 | 0.4523 | 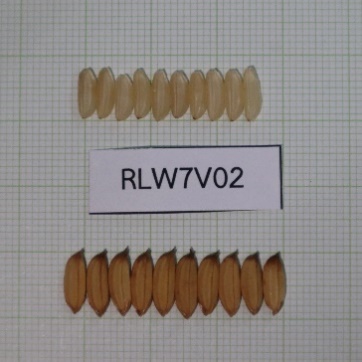 | |
| 20 | LV4 | RLW7V03 | เฮาะละทิง (นา) | Hao La Ting (Na) | Light | 6.250 | 3.001 | 2.107 | Medium | indica | 19266 | 7797 | 0.4047 | 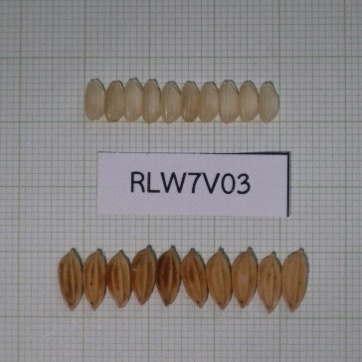 | |
| 21 | LV4 | RLW7V04 | เฮาะพิด (ไร่ เหนียว) | Hao Phit (Rai Niao) | Red | 6.991 | 2.111 | 3.301 | Slender | indica | 19736 | 8810 | 0.4464 | 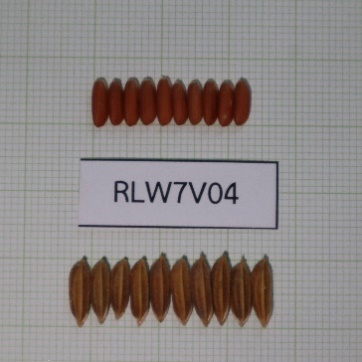 | |
| 22 | LV4 | RLW7V05 | เฮาะพุยช (ไร่ ลาย) | Hao Pui Ch (Rai Lai) | Light | 7.160 | 2.723 | 2.698 | Medium | japonica | 18848 | 1248 | 0.0662 | 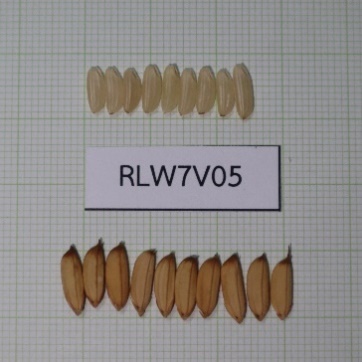 | |
| 23 | LV4 | RLW7V06 | เฮาะพิด (ไร่ เหนียว) | Hao Phit (Rai Niao) | Red | 6.873 | 2.336 | 3.019 | Slender | indica | 19726 | 8383 | 0.425 | 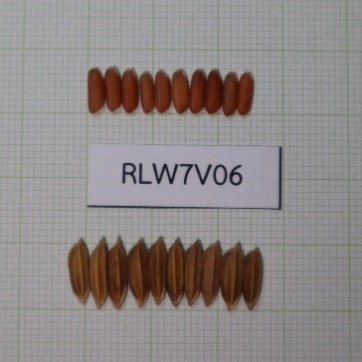 | |
| 24 | LV4 | RLW7V07 | เฮาะฉึกลองละขิด (นา เหนียว แข็ง) | Hao Chuk Long La Khit (Na Niao Khaeng) | Light | 7.938 | 2.446 | 3.272 | Slender | indica | 16912 | 7832 | 0.4631 | 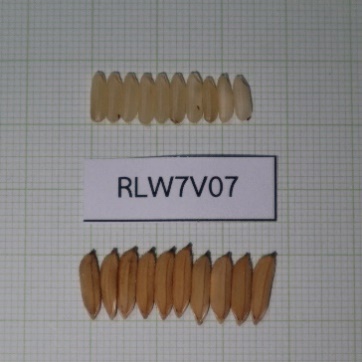 | |
| 25 | LV4 | RLW7V08 | เฮาะละวง (ไร่ เจ้า) | Hao La Wong (Rai Chao) | Light | 6.869 | 2.958 | 2.328 | Medium | japonica | 15927 | 947 | 0.0595 | 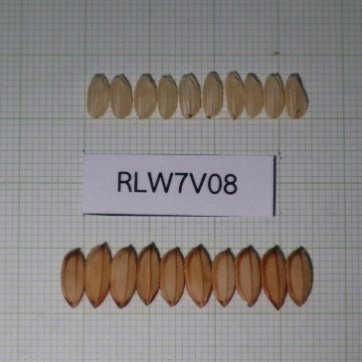 | |
| 26 | LV4 | RLW7V09 | เฮาะฉึก (นา เจ้า) | Hao Chuk (Na Chao) | Off-white | 7.595 | 2.957 | 2.517 | Medium | indica | 17881 | 7214 | 0.4034 | 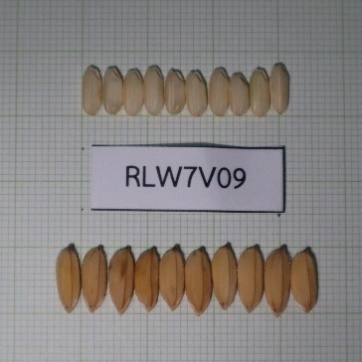 | |
| 27 | LV4 | RLW7V10 | เฮาะละทิง (นา เหนียว) | Hao La Ting (Na Niao) | Off-white | 6.643 | 3.044 | 2.249 | Medium | indica | 17491 | 7005 | 0.4005 | 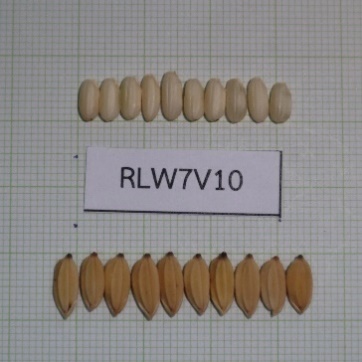 | |
| 28 | DA1 | RLW5V01 | ข้าวก่ำ | Khao Kam | Black | 8.162 | 2.841 | 2.800 | Medium | japonica | 51343 | 2227 | 0.0434 | 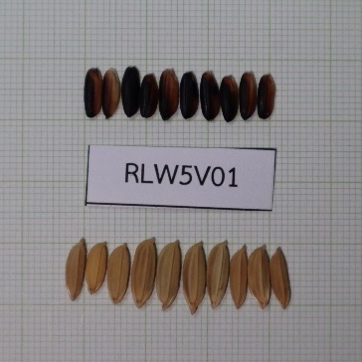 | |
| 29 | DA1 | RLW5V02 | ข้าวแป | Khao Pae | Light | 7.503 | 3.077 | 2.405 | Medium | japonica | 27795 | 2064 | 0.0743 | 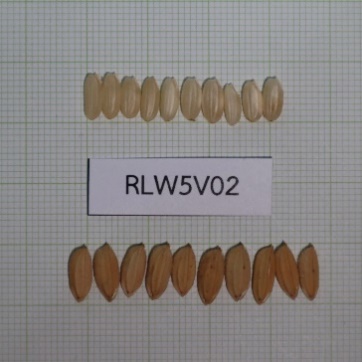 | |
| 30 | DA1 | RLW5V05 | เหนียวดำ (ข้าวแซ่ง) | Niao Dam (Khao Saeng) | Black | 8.469 | 2.900 | 2.880 | Medium | japonica | 46814 | 2146 | 0.0458 | 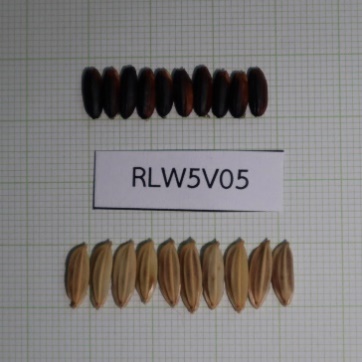 | |
| 31 | DA1 | RLW5V06 | ข้าวเจ้า (คุณลุงชุ) | Khao Chao (Khun Lung Chu) | Light | 7.329 | 2.953 | 2.446 | Medium | indica | 47889 | 13665 | 0.2853 | 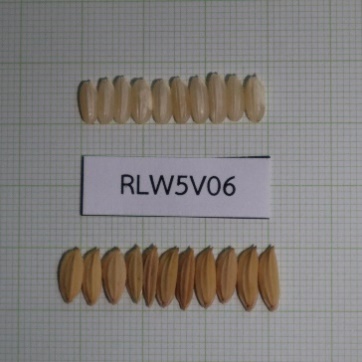 | |
| 32 | DA1 | RLW5V07 | ข้าวเจ้า (ปาหว่อง) | Khao Chao (Pa Wong) | Light | 6.933 | 3.272 | 2.077 | Medium | japonica | 49216 | 2648 | 0.0538 | 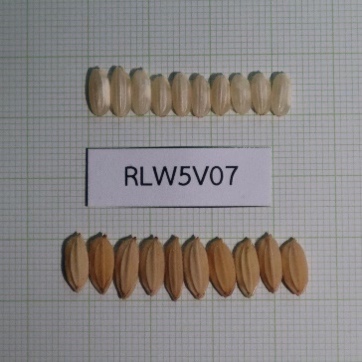 | |
| 33 | DA1 | RLW5V09 | ข้าวเหนียว (โคนงซู) | Khao Niao (Konong Su) | Red | 6.557 | 2.205 | 2.996 | Medium | indica | 47344 | 13353 | 0.282 | 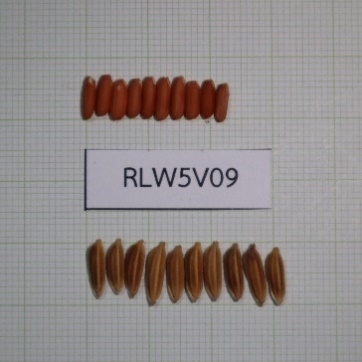 | |
| 34 | DA1 | RLW5V11 | ข้าวเจ้าหอม (เฮาแรน) | Khao Chao Hom (Hao Ran) | Light | 6.651 | 2.650 | 2.629 | Medium | indica | 47568 | 12356 | 0.2598 | 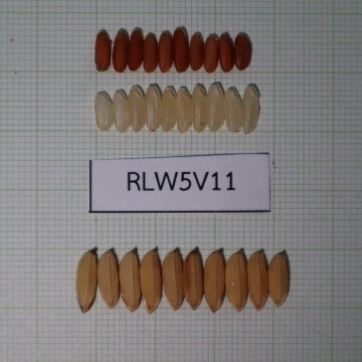 | |
| 35 | DA1 | RLW5V14 | ข้าวเหนียว ข้าวก่ำ ข้าวเซ | Khao Niao, Khao Kam, Khao Se | Black | 8.020 | 2.660 | 2.950 | Medium | japonica | 47023 | 2214 | 0.0471 | 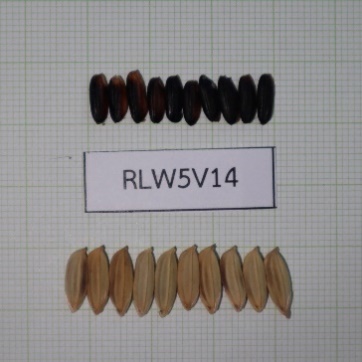 | |
| 36 | DA1 | RLW5V15 | ข้าวดอย | Khao Doi | Light | 7.562 | 3.434 | 2.219 | Medium | japonica | 47693 | 2786 | 0.0584 | 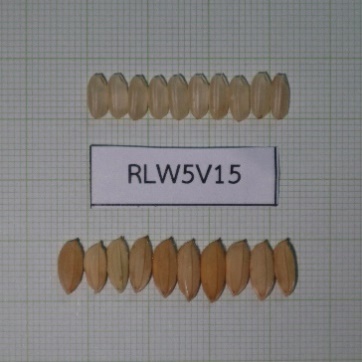 | |
| 37 | DA1 | RLW5V17 | ข้าวเหนียวดอย (ข้าวแดง) | Khao Niao Doi (Khao Daeng) | Off-white | 7.947 | 2.955 | 2.697 | Medium | indica | 45583 | 12535 | 0.275 | 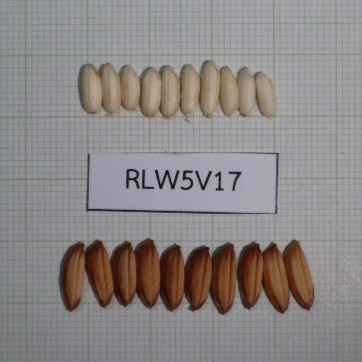 | |
| 38 | DA1 | RLW5V20 | ข้าวเจ้า (ปะล้ว) | Khao Chao (Pa Lue) | Light | 7.155 | 3.221 | 2.260 | Medium | japonica | 47151 | 2204 | 0.0467 | 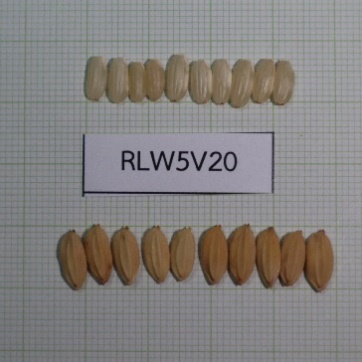 | |
| 39 | DA1 | RLW5V22 | ข้าวลาย | Khao Lai | Light | 8.127 | 2.615 | 3.208 | Slender | japonica | 45485 | 2382 | 0.0524 | 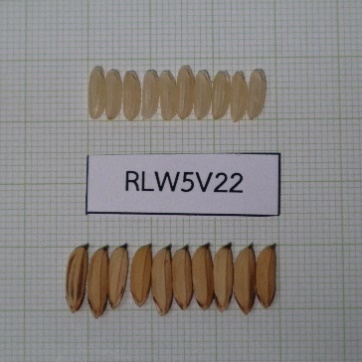 | |
| 40 | DA1 | RLW5V24 | ข้าวเจ้า | Khao Chao | Light | 7.617 | 3.077 | 2.468 | Medium | indica | 43365 | 11064 | 0.2551 | 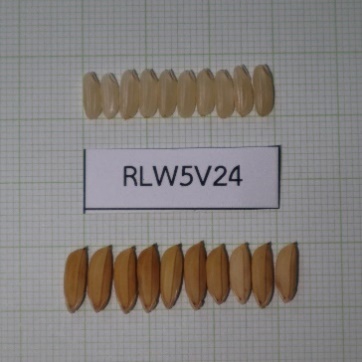 | |
| 41 | DA1 | RLW5V28 | ข้าวดอย (เมล็ดน้ำผึ้ง) | Khao Doi (Malet Nam Phueng) | Light | 7.672 | 2.808 | 2.627 | Medium | japonica | 43222 | 2661 | 0.0616 | 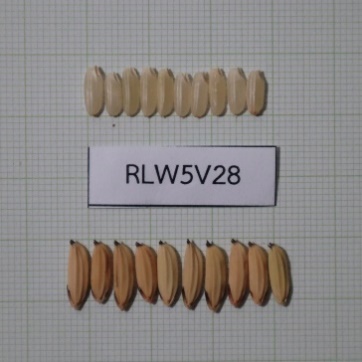 | |
| 42 | DA1 | RLW5V32 | ตองซู | Tong Su | Light | 6.915 | 3.192 | 2.143 | Medium | japonica | 26448 | 1945 | 0.0735 | 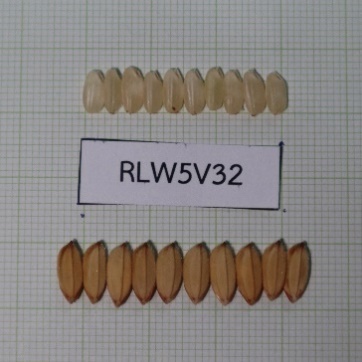 | |
| 43 | DA1 | RLW5V34 | ปะว้อ | Pa Wor | Light | 8.032 | 2.556 | 3.207 | Slender | japonica | 43775 | 2281 | 0.0521 | 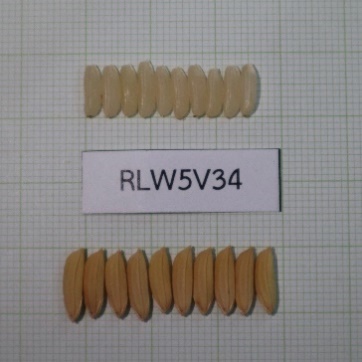 | |
| 44 | LW1 | RLW6V01 | ข้าวไร่ (ข้าวเจ้าผสมข้าวเหนียว) | Khao Rai (Khao Chao Phasom Khao Niao) | Red | 6.225 | 2.652 | 2.367 | Medium | indica | 42088 | 10968 | 0.2606 | 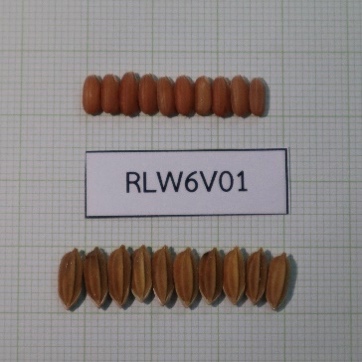 | |
| 45 | LW1 | RLW6V02 | ข้าวกลาง (ข้าวเจ้า) | Khao Klang (Khao Chao) | Light | 7.563 | 2.868 | 2.584 | Medium | japonica | 42137 | 2146 | 0.0509 | 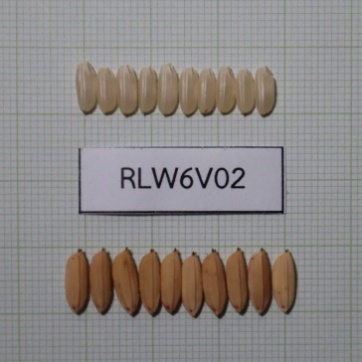 | |
| 46 | LW1 | RLW6V03 | ข้าวไร่ (ข้าวเจ้า) | Khao Rai (Khao Chao) | Light | 7.127 | 2.688 | 2.776 | Medium | japonica | 41124 | 2137 | 0.052 | 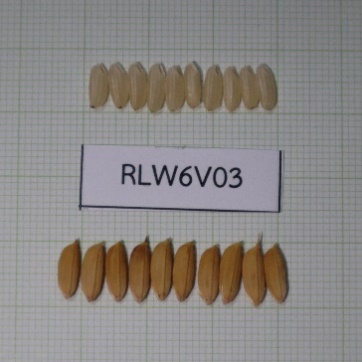 | |
| 47 | LW1 | RLW6V04 | ข้าวเจ้า (ดำ) | Khao Chao (Dam) | Light | 6.925 | 2.438 | 2.820 | Medium | japonica | 41825 | 2222 | 0.0531 | 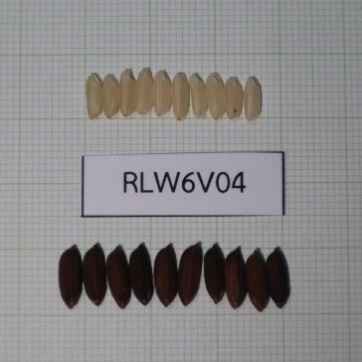 | |
| 48 | LW1 | RLW6V05 | ข้าวเจ้า | Khao Chao | Light | 8.357 | 2.797 | 3.077 | Slender | japonica | 39254 | 2078 | 0.0529 | 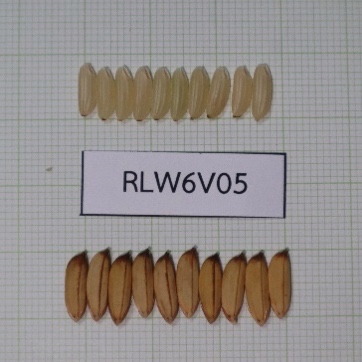 | |
| 49 | LW1 | RLW6V06 | ข้าวเหนียว (แดงนิดๆ) | Khao Niao (Daeng Nit Nit) | Red | 6.408 | 2.223 | 2.949 | Medium | indica | 37526 | 11000 | 0.2931 | 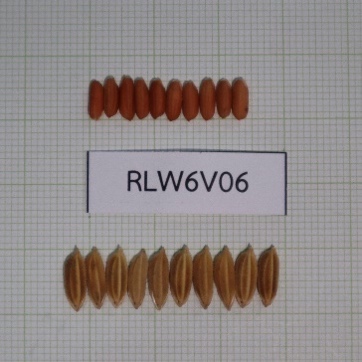 | |
| 50 | LW1 | RLW6V07 | ข้าวลีซอ | Khao Lisu | Light | 7.784 | 2.631 | 2.942 | Medium | japonica | 36803 | 2026 | 0.055 | 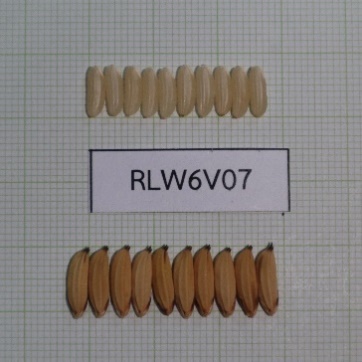 | |
| 51 | LW1 | RLW6V08 | ข้าวนา (ข้าวเจ้า) | Khao Na (Khao Chao) | Light | 6.223 | 3.112 | 1.953 | Bold | indica | 35709 | 10048 | 0.2814 | 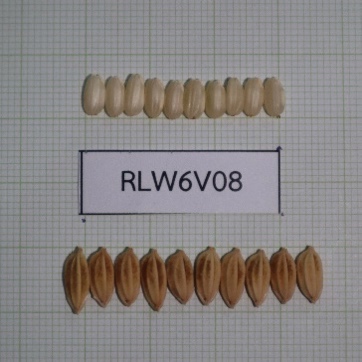 | |
| 52 | KM1 | RKU1V01 | ข้าวแม้ว | Khao Maew | Off-white | 7.319 | 3.262 | 2.314 | Medium | japonica | 33418 | 2340 | 0.07 | 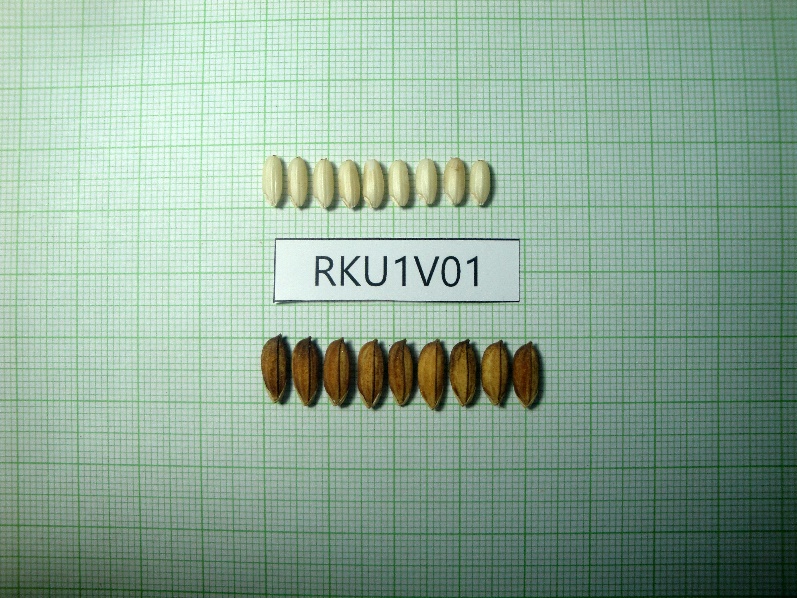 | |
| 53 | KM1 | RKU1V03 | ข้าวลาย | Khao Lai | Off-white | 7.730 | 3.059 | 2.458 | Medium | japonica | 36345 | 2492 | 0.0686 | 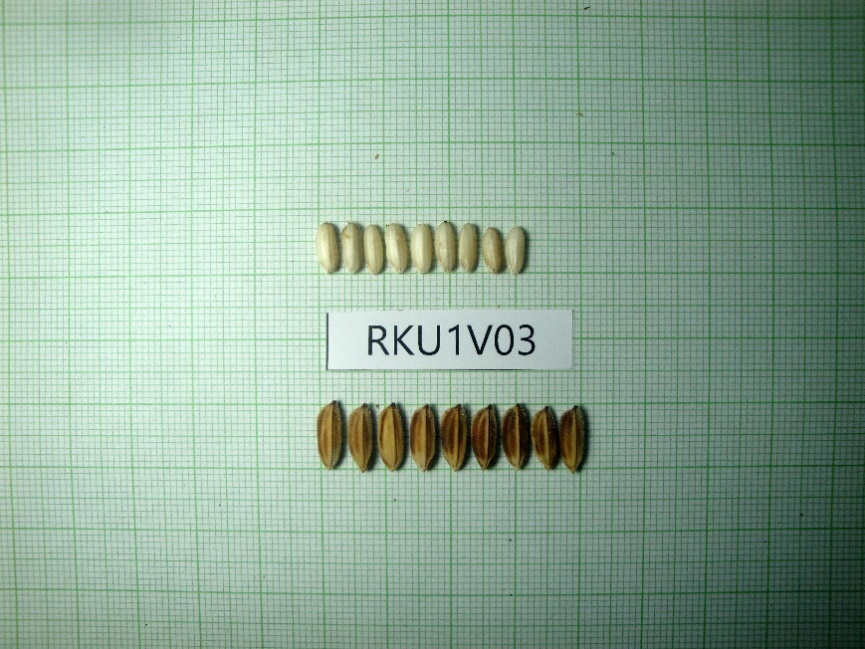 | |
| 54 | KM1 | RKU1V04 | ข้าวแดงกาย | Khao Daeng Kai | Off-white | 6.497 | 3.374 | 1.937 | Bold | japonica | 33351 | 1889 | 0.0566 | 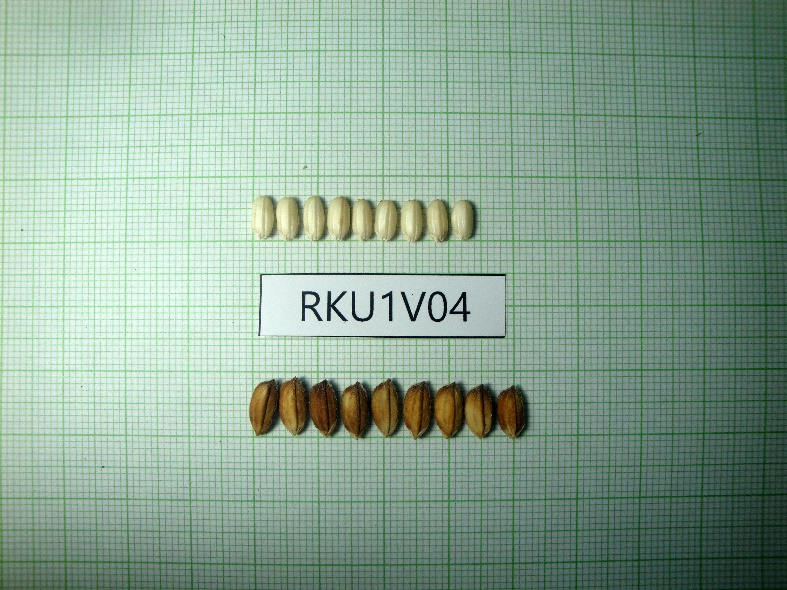 | |
| 55 | KM1 | RKU1V05 | ข้าวแดง(เหนียว) | Khao Daeng (Niao) | Off-white | 7.115 | 3.159 | 2.305 | Medium | japonica | 30860 | 1923 | 0.0623 | 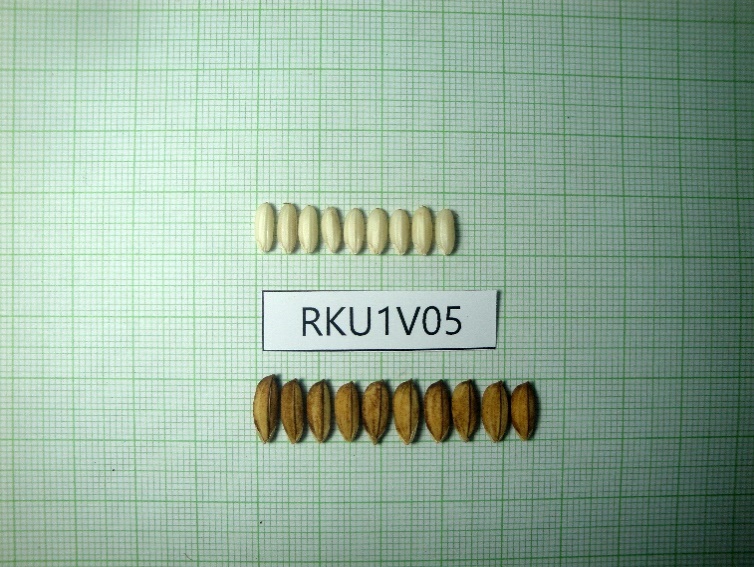 | |
| 56 | KM1 | RKU1V07 | ข้าวแดง | Khao Daeng | Off-white | 7.286 | 3.013 | 2.344 | Medium | japonica | 30652 | 2015 | 0.0657 | **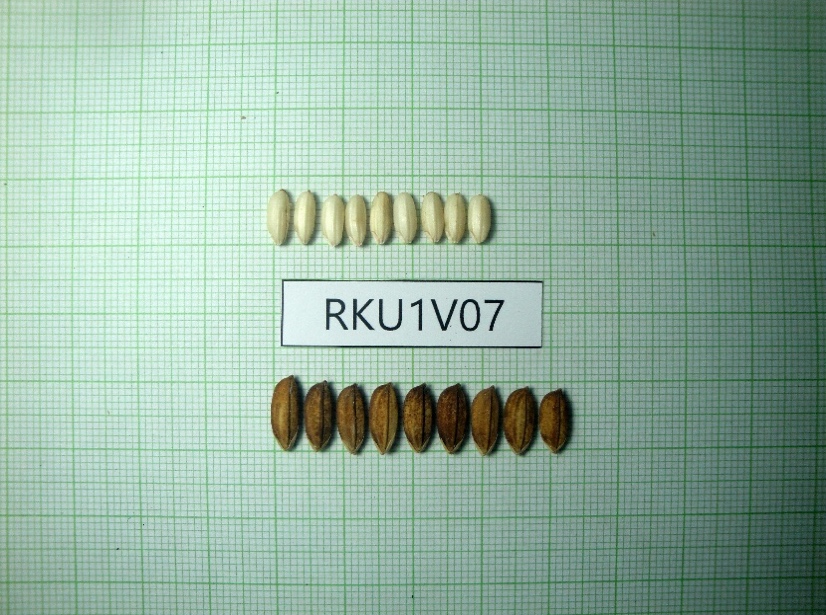** | |
| 57 | KM1 | RKU1V09 | ข้าวขาว | Khao Khao | Off-white | 7.392 | 3.228 | 2.261 | Medium | japonica | 33373 | 2001 | 0.06 | **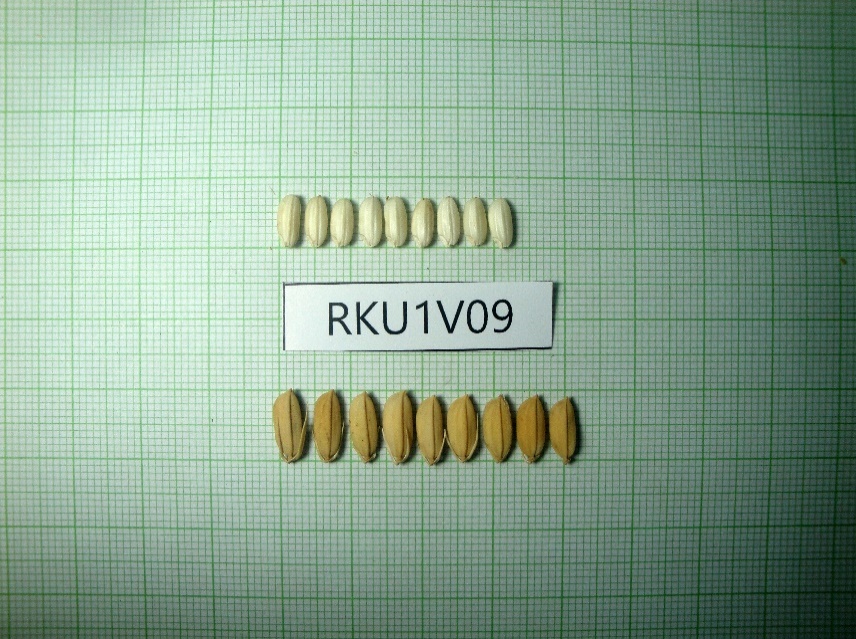** | |
| 58 | KM1 | RKU1V13 | ข้าวแดงหลวง | Khao Daeng Luang | Off-white | 8.023 | 3.182 | 2.473 | Medium | indica | 35147 | 12179 | 0.3465 | 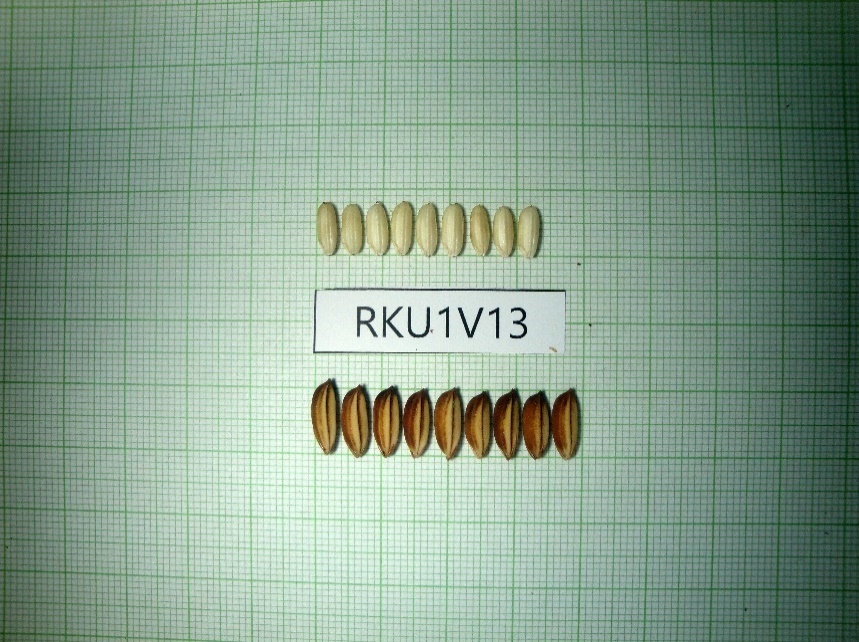 | |
| 59 | KM1 | RKU1V19 | ข้าวขาว | Khao Khao | Off-white | 8.373 | 3.111 | 2.685 | Medium | japonica | 32722 | 2086 | 0.0637 | **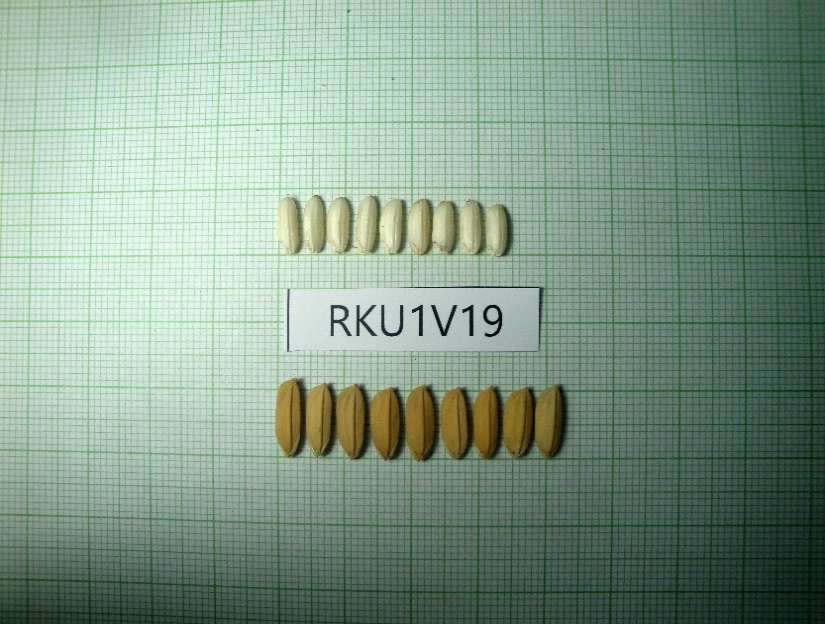** | |
| 60 | KM1 | RKU1V20 | ข้าวแดงเหนียว | Khao Daeng Niao | Off-white | 7.195 | 3.038 | 2.376 | Medium | japonica | 24646 | 1539 | 0.0624 | **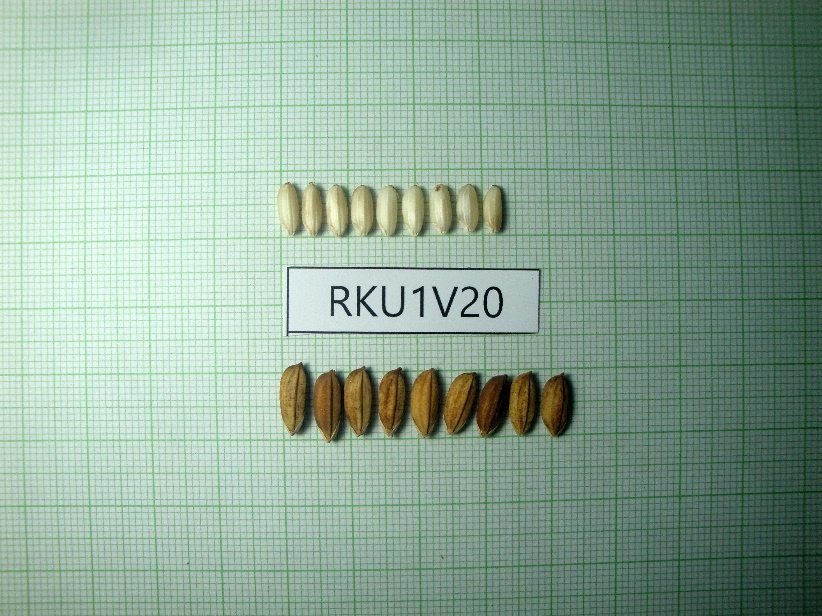** | |
| 61 | KM1 | RKU1V23 | ข้าวขาวเหนียวใหญ่ | Khao Khao Niao Yai | Off-white | 7.707 | 3.035 | 2.543 | Medium | japonica | 31817 | 2296 | 0.0722 | **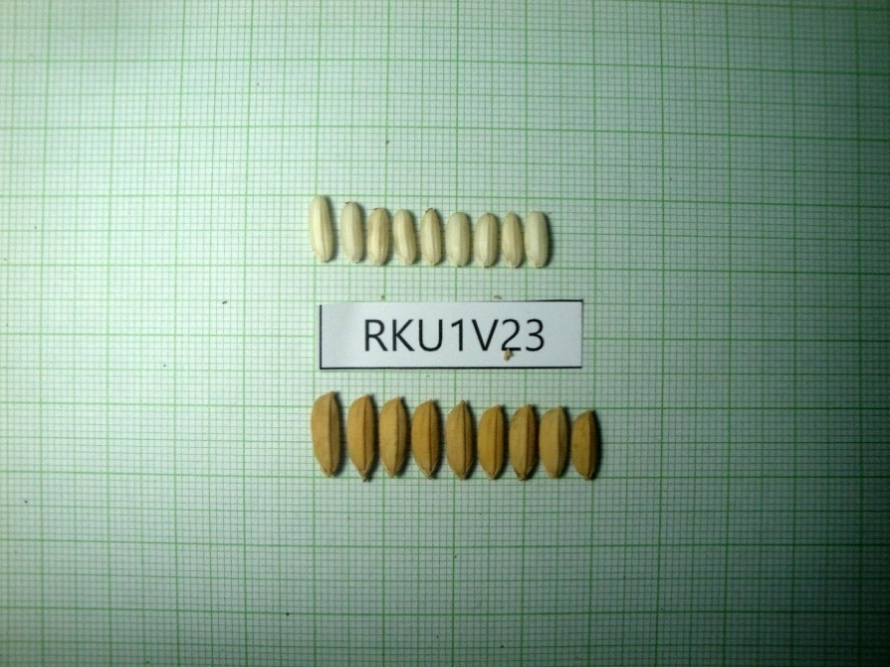** | |
| 62 | KM2 | RKU2V01 | ข้าวเจ้าพื้นเมือง | Khao Chao Phuen Mueang | Light | 7.174 | 2.622 | 2.696 | Medium | japonica | 35155 | 2455 | 0.0698 | 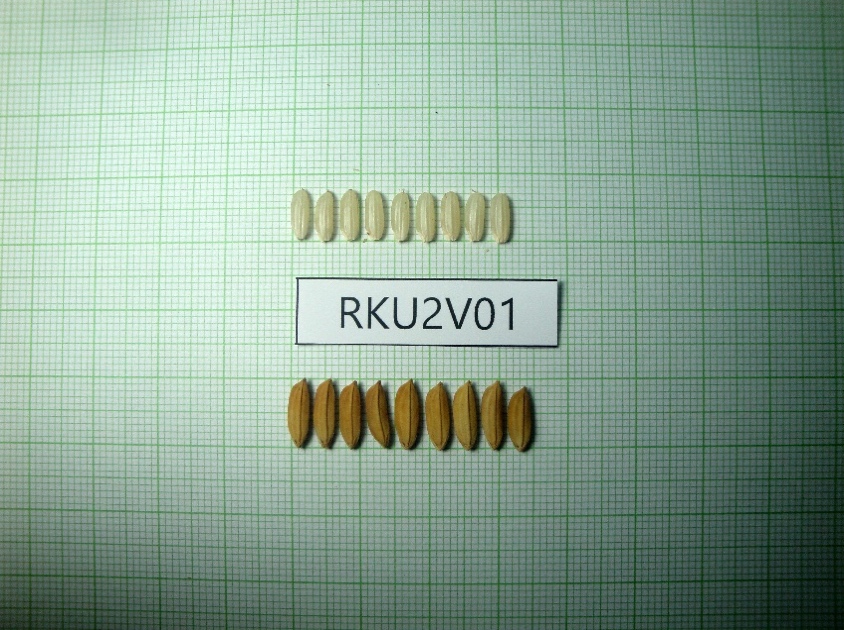 | |
| 63 | KM2 | RKU2V02 | ข้าวขาวเม็ดใหญ่ | Khao Khao Met Yai | Off-white | 8.261 | 2.941 | 2.846 | Medium | japonica | 28540 | 2066 | 0.0724 | **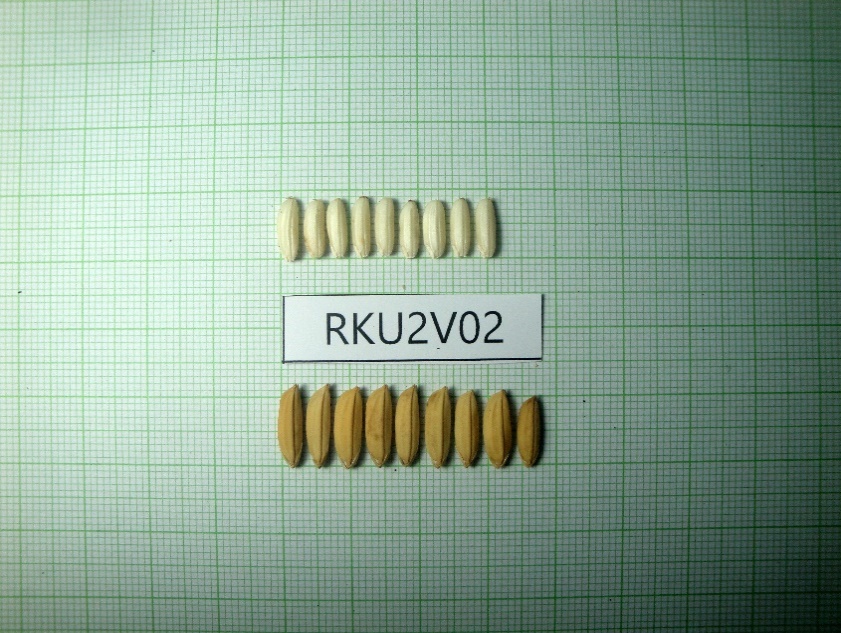** | |
| 64 | KM2 | RKU2V03 | ข้าวก่ำเมล็ดยาว | Khao Kam Malet Yao | Black | 8.043 | 3.048 | 2.602 | Medium | indica | 33099 | 12621 | 0.3813 | **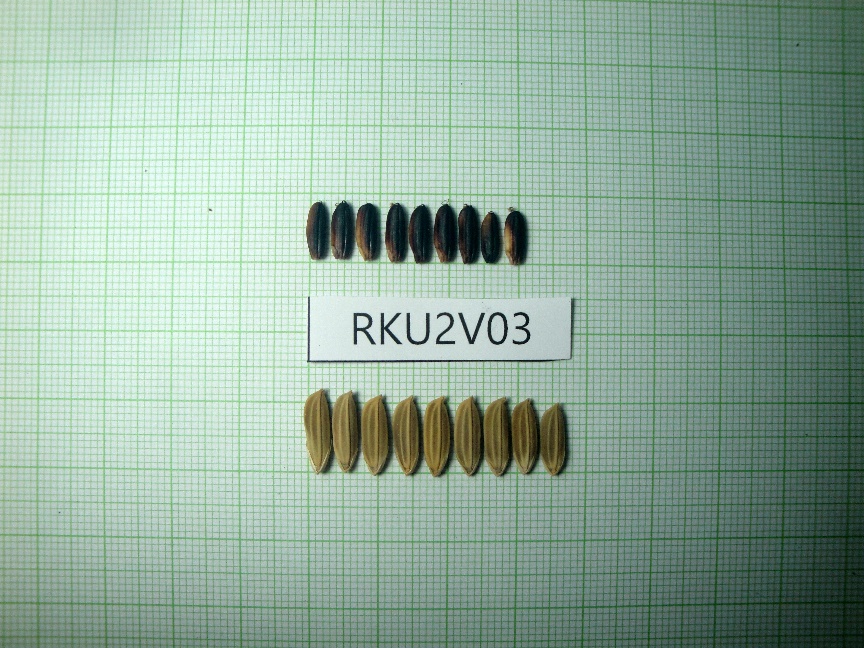** | |
| 65 | KM2 | RKU2V04 | ข้าวก่ำเมล็ดสั้น | Khao Kam Malet San | Black | 6.960 | 3.316 | 2.076 | Medium | japonica | 28672 | 1594 | 0.0556 | **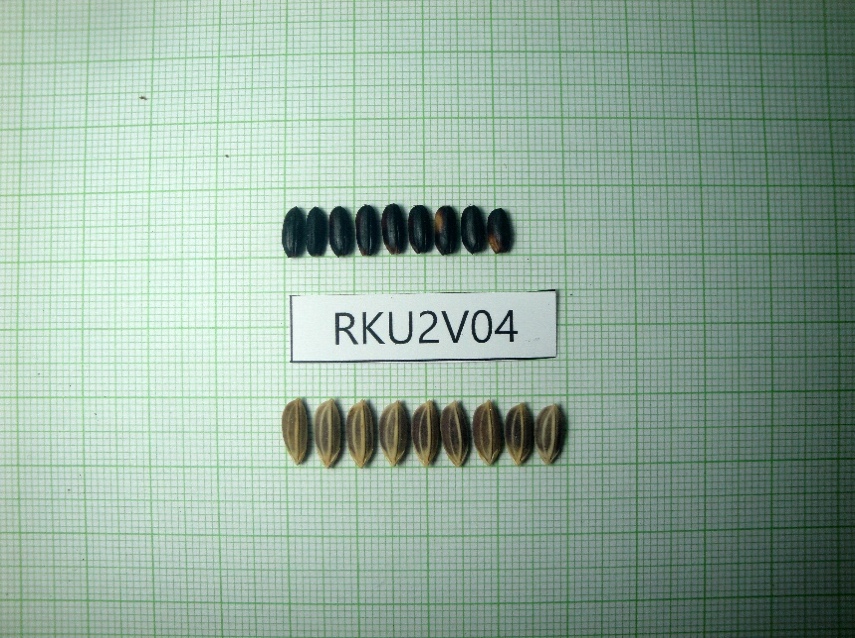** | |
| 66 | KM2 | RKU2V06 | ข้าวหนอนขาว | Khao Non Khao | Off-white | 8.254 | 2.991 | 2.815 | Medium | indica | 32518 | 16275 | 0.5005 | **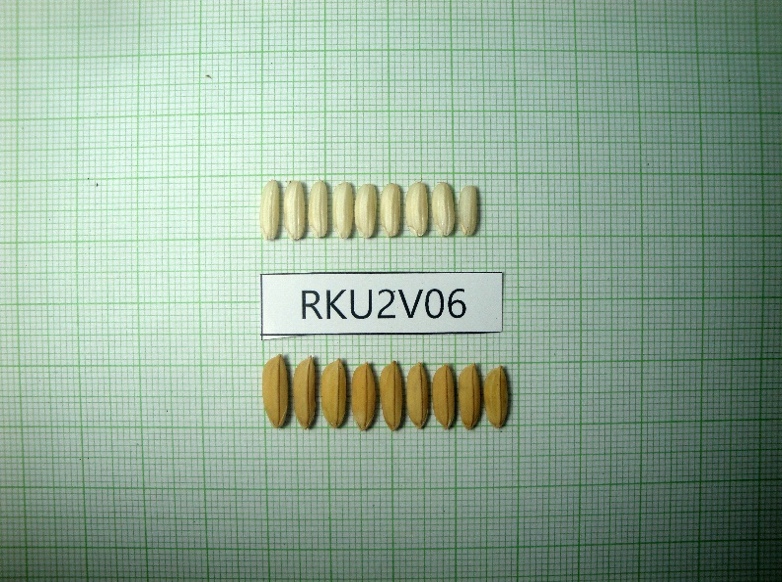** | |
| 67 | KM2 | RKU2V08 | ข้าวก่ำยาวเหนียว | Khao Kam Yao Niao | Black | 8.383 | 2.555 | 3.273 | Slender | japonica | 34329 | 1765 | 0.0514 | 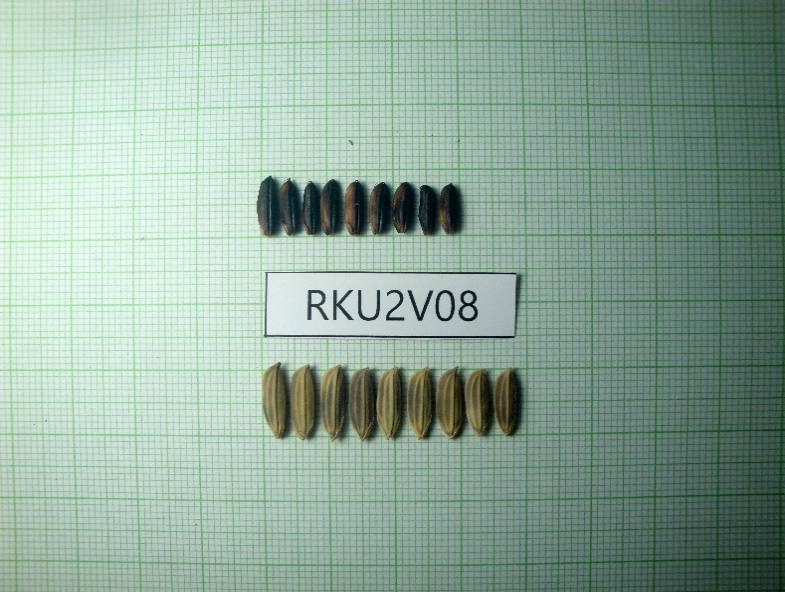 | |
| 68 | KM2 | RKU2V09 | ข้าวกองพาย | Khao Kong Pai | Off-white | 9.649 | 2.991 | 3.224 | Slender | japonica | 36616 | 2336 | 0.0638 | **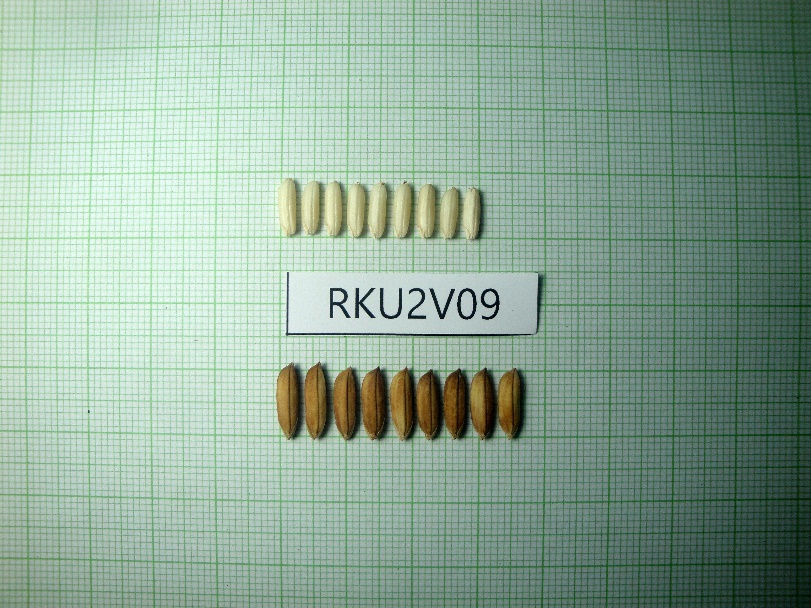** | |
| 69 | KM2 | RKU2V10 | ข้าวขาวหอม | Khao Khao Hom | Off-white | 6.932 | 3.260 | 2.127 | Medium | japonica | 25856 | 1831 | 0.0708 | 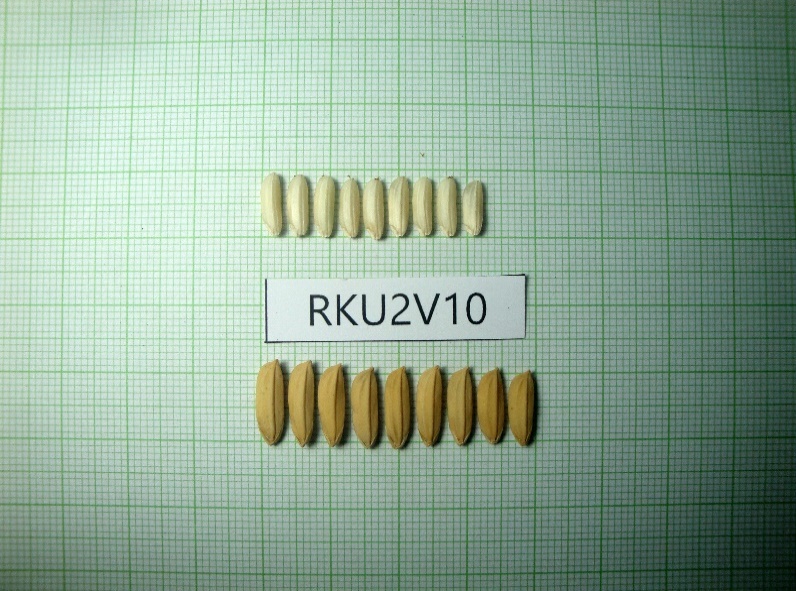 | |
| 70 | KM2 | RKU2V12 | ข้าวขาวเหนียว | Khao Khao Niao | Off-white | 8.690 | 3.293 | 2.799 | Medium | japonica | 24888 | 2044 | 0.0821 | **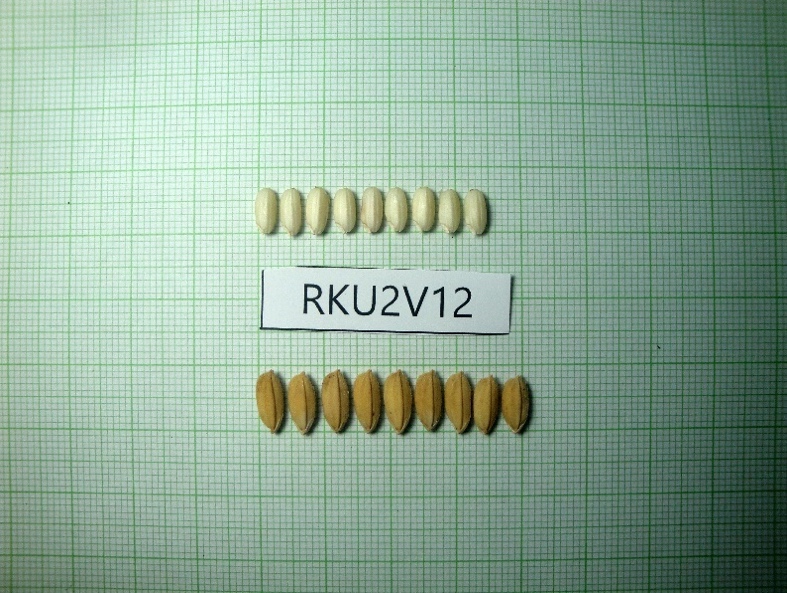** | |
| 71 | KM3 | RKU3V02 | ข้าวแดงยาว | Khao Daeng Yao | Off-white | 8.317 | 3.055 | 2.734 | Medium | japonica | 28817 | 1453 | 0.0504 | **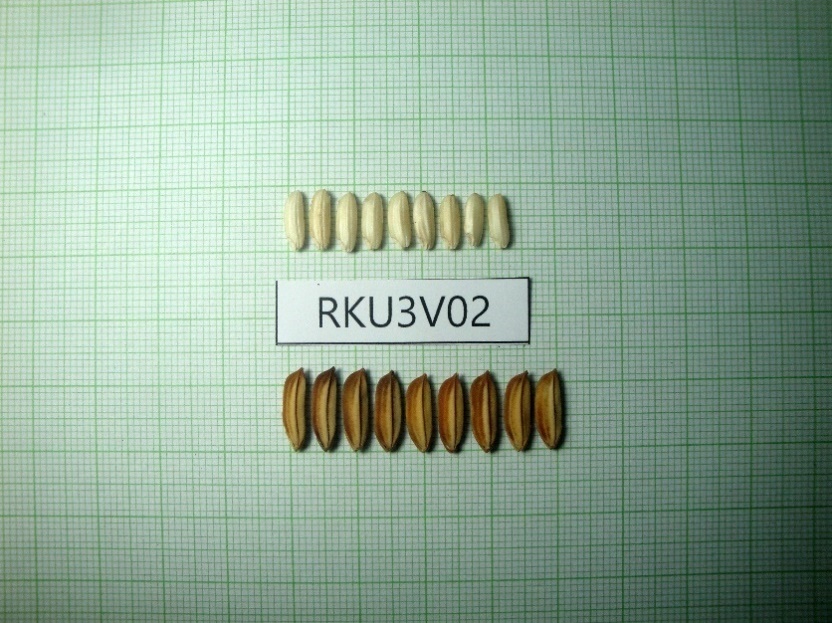** | |
| 72 | KM3 | RKU3V03 | ข้าวก่ำครั่ง | Khao Kam Khrang | Black | 7.978 | 3.056 | 2.570 | Medium | indica | 33715 | 16540 | 0.4906 | **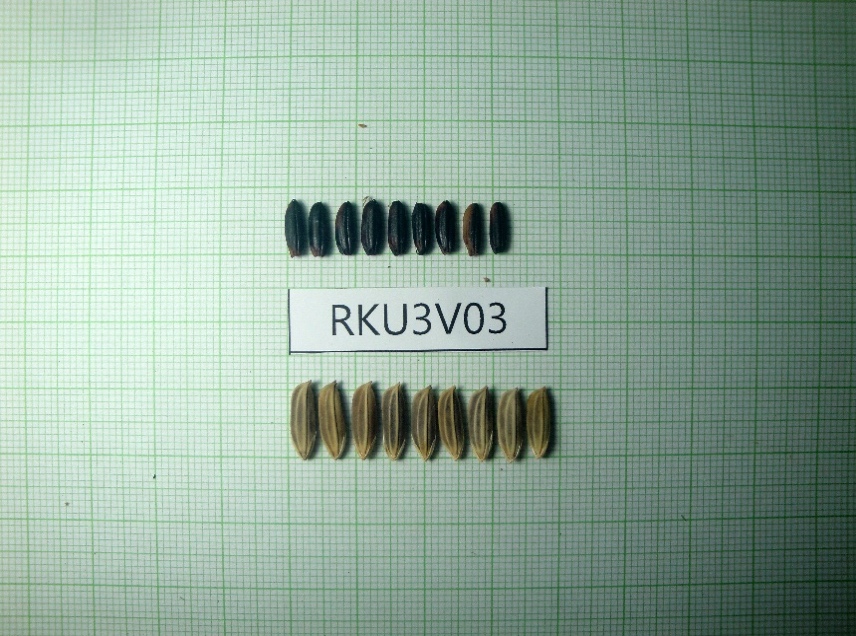** | |
| 73 | KM3 | RKU3V04 | ข้าวแดง | Khao Daeng | Off-white | 8.763 | 3.221 | 2.691 | Medium | japonica | 31708 | 2045 | 0.0645 | **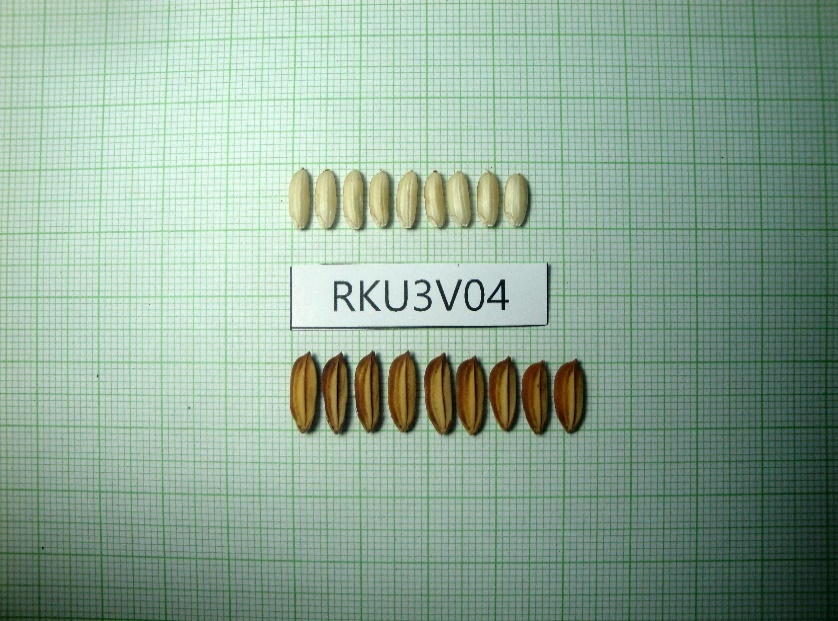** | |
| 74 | KM3 | RKU3V05 | ข้าวดำ | Khao Dam | Black | 8.315 | 2.943 | 2.802 | Medium | japonica | 30721 | 2002 | 0.0652 | **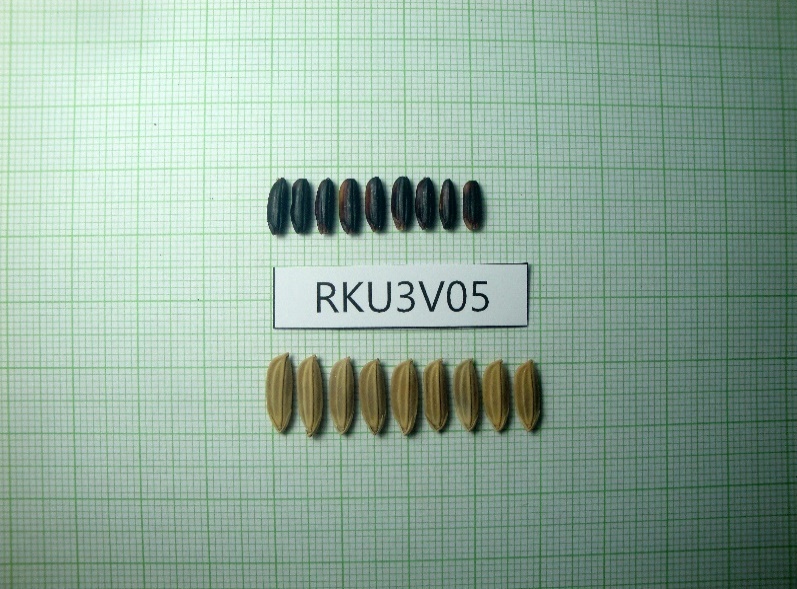** | |
| 75 | KM3 | RKU3V08 | ข้าวก่ำแท้ | Khao Kam Tae | Black | 8.241 | 2.847 | 2.810 | Medium | japonica | 30788 | 1818 | 0.059 | **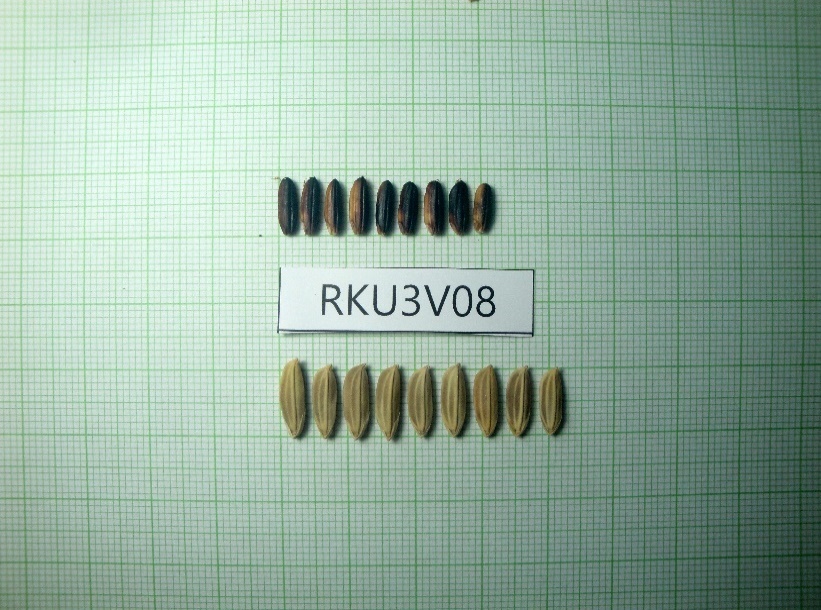** | |
| 76 | KM3 | RKU3V09 | ข้าวแดงกลาง | Khao Daeng Klang | Off-white | 7.035 | 3.308 | 2.196 | Medium | japonica | 31811 | 2047 | 0.0643 | **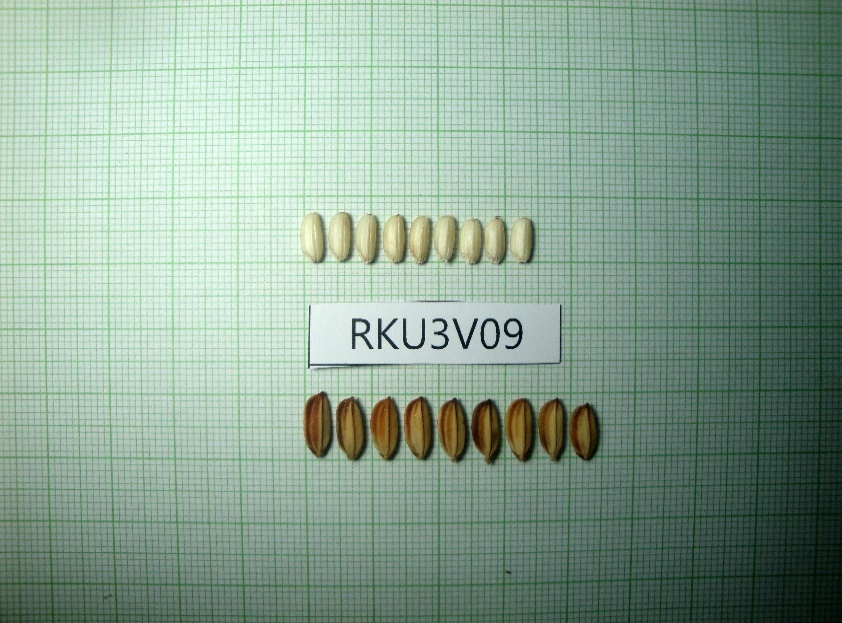** | |
| 77 | KM3 | RKU3V10 | ข้าวควายหาย | Khao Khwai Hai | Off-white | 8.570 | 3.155 | 2.677 | Medium | japonica | 33514 | 1844 | 0.055 | **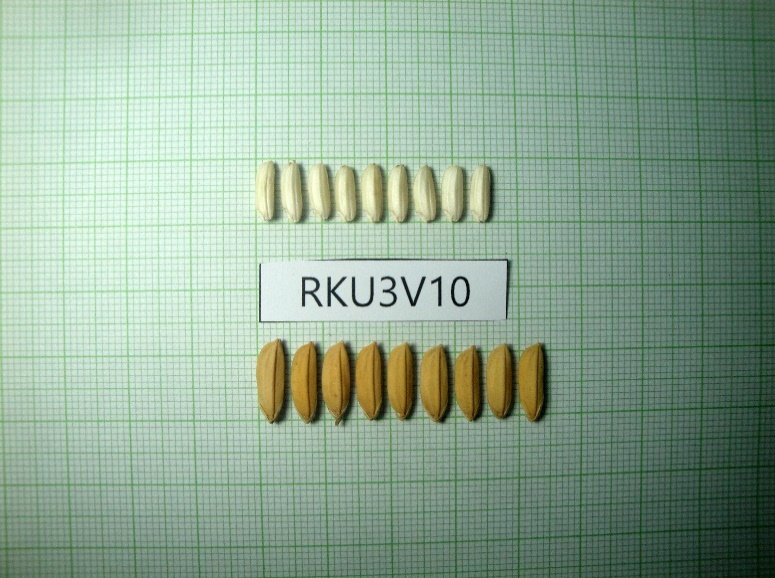** | |
| 78 | KM3 | RKU3V14 | ข้าวซิว | Khao Siw | White | 7.778 | 2.208 | 3.677 | Slender | indica | 32241 | 12344 | 0.3829 | **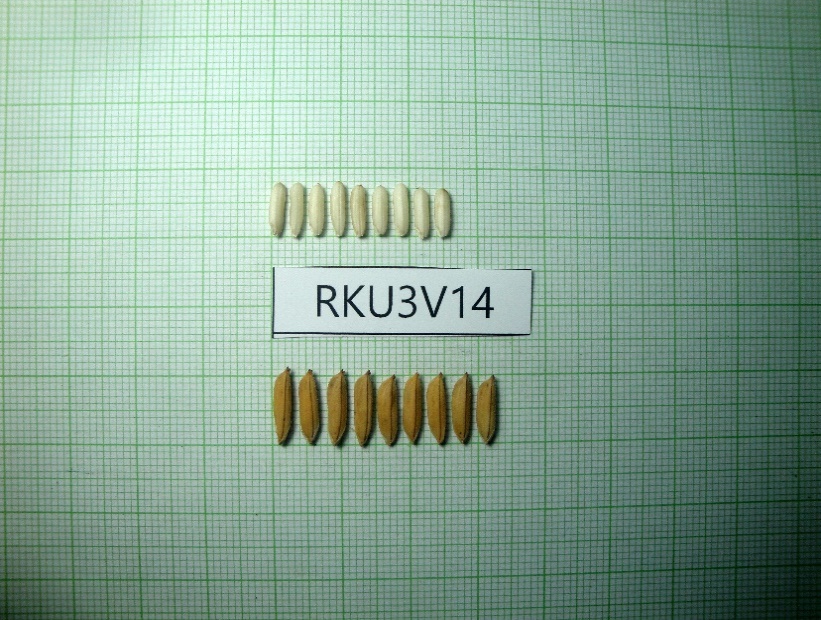** | |
| 79 | KM3 | RKU3V17 | ข้าวกุน | Khao Kun | Off-white | 6.077 | 3.110 | 1.999 | Bold | japonica | 29472 | 1478 | 0.0501 | **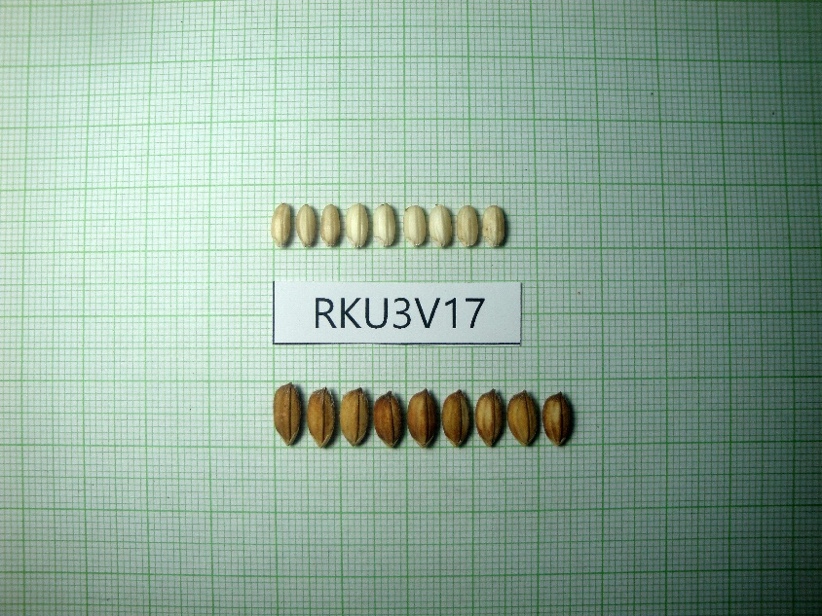** | |
| 80 | KM3 | RKU3V22 | ข้าวควายหาย | Khao Khwai Hai | Off-white | 9.303 | 3.089 | 2.972 | Medium | japonica | 27060 | 1569 | 0.058 | 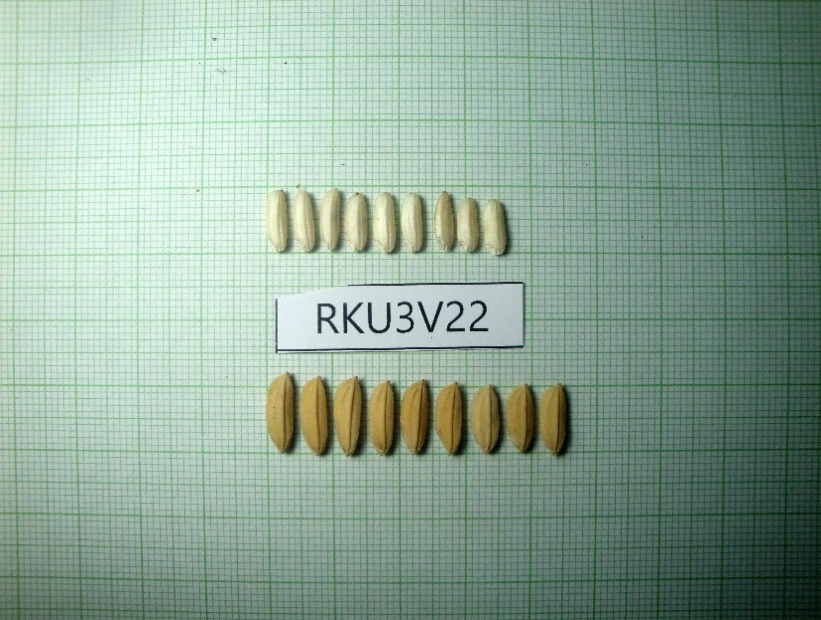 | |
| 81 | LU1 | RLA1V02 | ข้าวก่ำดำ | Khao Kam Dam | Black | 7.274 | 3.166 | 2.405 | Medium | japonica | 54475 | 2451 | 0.045 | **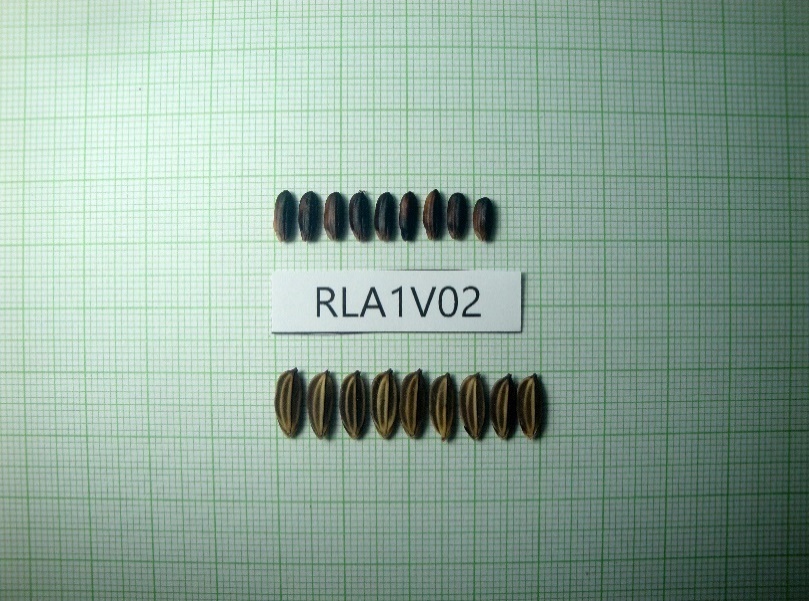** | |
| 82 | LU1 | RLA1V03 | ข้าวกุก | Khao Kuk | Off-white | 7.044 | 3.334 | 2.077 | Medium | japonica | 31204 | 1920 | 0.0615 | **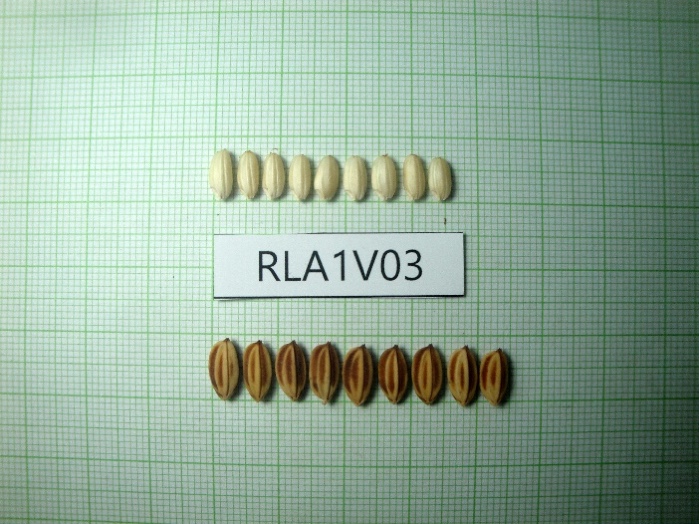** | |
| 83 | LU1 | RLA1V04 | ข้าวก่ำ | Khao Kam | Black | 6.832 | 3.223 | 2.077 | Medium | japonica | 24827 | 1726 | 0.0695 | **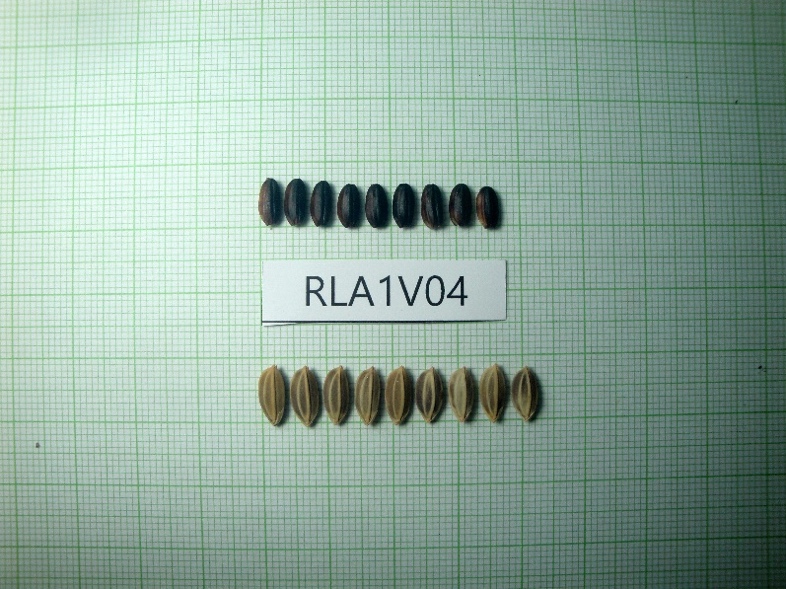** | |
| 84 | LU1 | RLA1V05 | ข้าวหอมอ้ม | Khao Hom Om | Brown | 7.725 | 3.391 | 2.250 | Medium | japonica | 28437 | 2143 | 0.0754 | **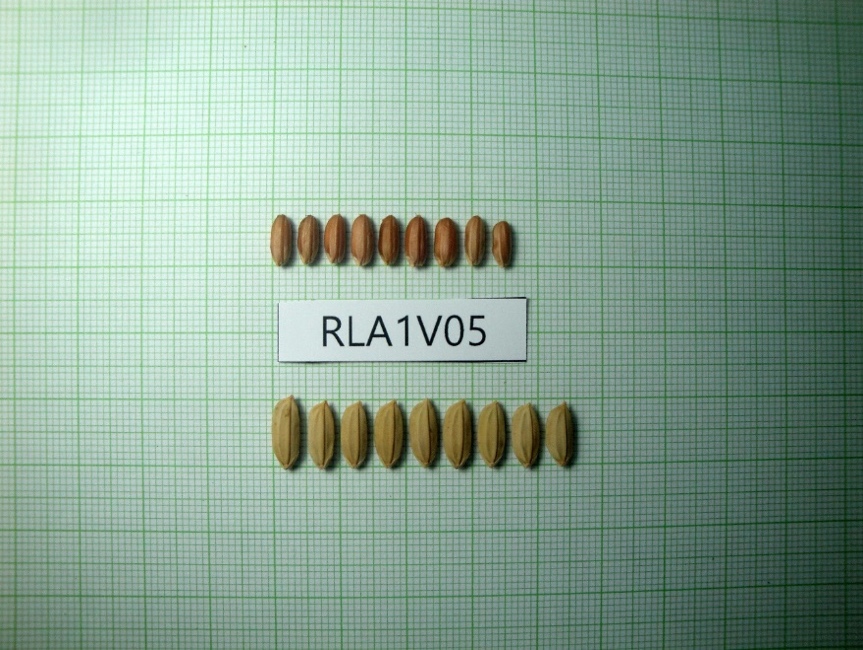** | |
| 85 | LU1 | RLA1V07 | ข้าวดำ | Khao Dam | Off-white | 8.407 | 3.334 | 2.497 | Medium | japonica | 57499 | 2489 | 0.0433 | **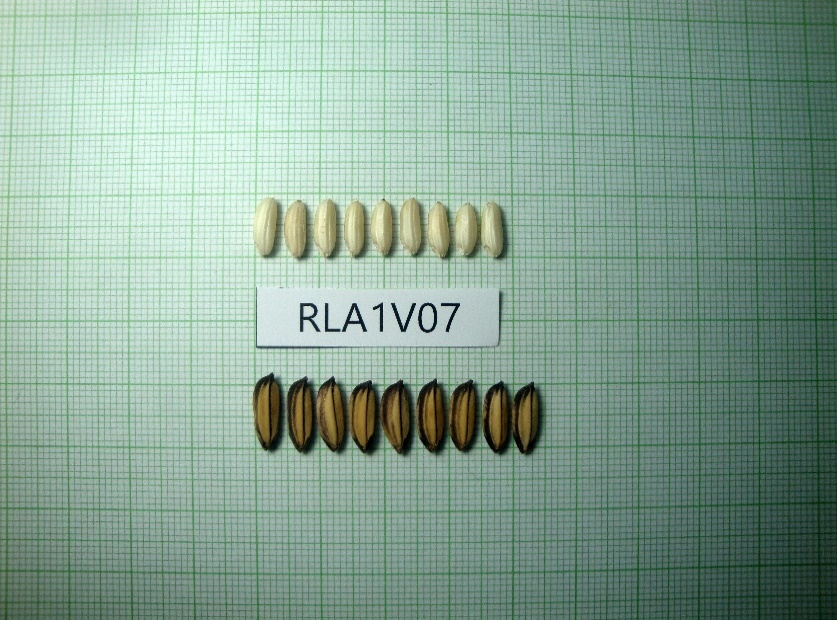** | |
| 86 | LU1 | RLA1V08 | ข้าวก่ำ | Khao Kam | Black | 7.512 | 3.100 | 2.493 | Medium | japonica | 57125 | 2343 | 0.041 | **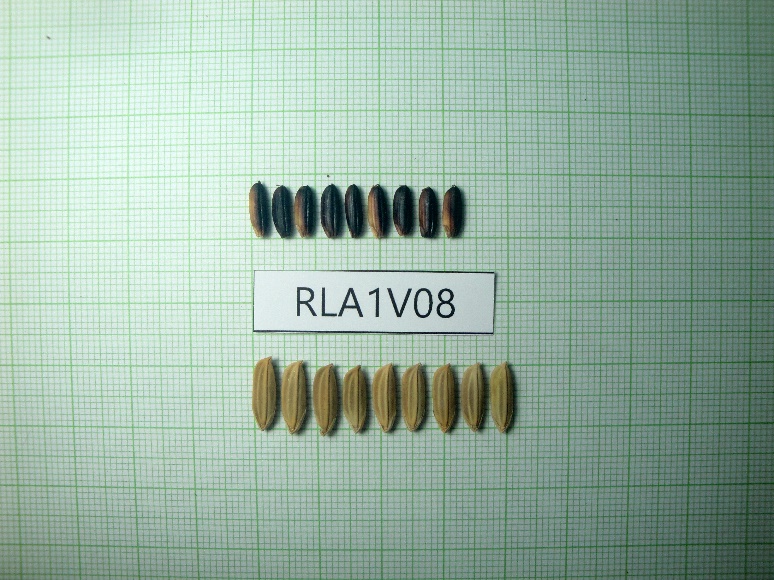** | |
| 87 | LU1 | RLA1V12 | ข้าวลาย | Khao Lai | Off-white | 8.345 | 2.972 | 2.864 | Medium | japonica | 54929 | 2592 | 0.0472 | **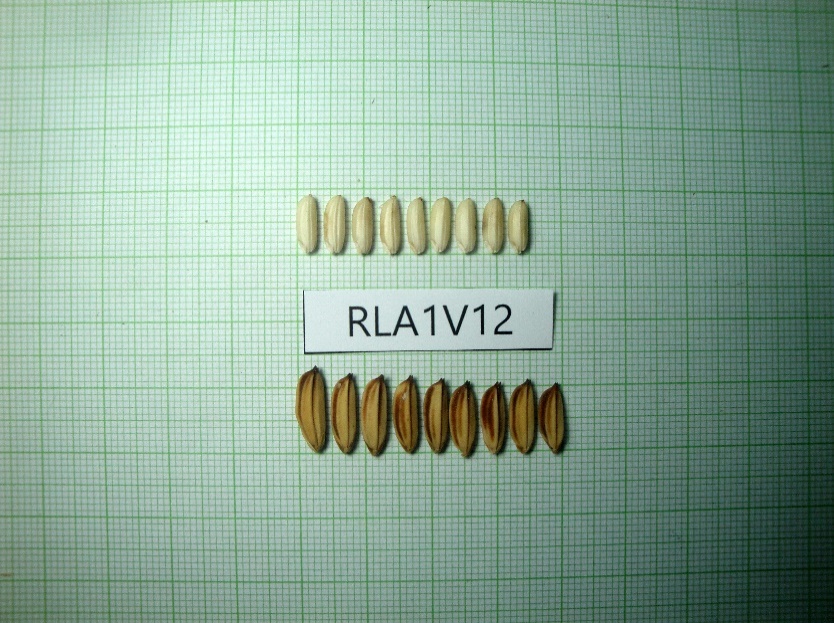** | |
| 88 | LU1 | RLA1V13 | ข้าวแก่นฝ้าย | Khao Kaen Fai | Off-white | 7.556 | 3.044 | 2.480 | Medium | japonica | 56368 | 2935 | 0.0521 | **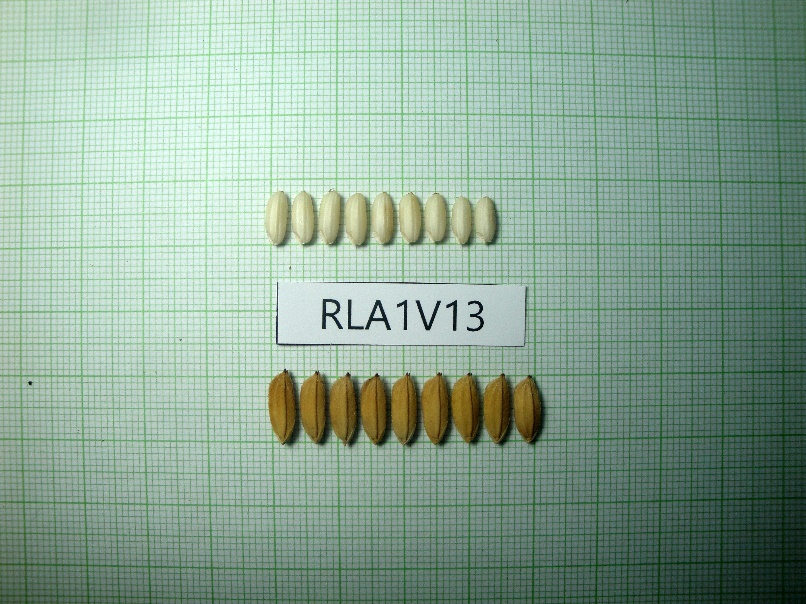** | |
| 89 | LU1 | RLA1V16 | ข้าวฝักดาบ | Khao Fak Dab | Off-white | 8.688 | 3.130 | 2.750 | Medium | japonica | 31073 | 1925 | 0.062 | **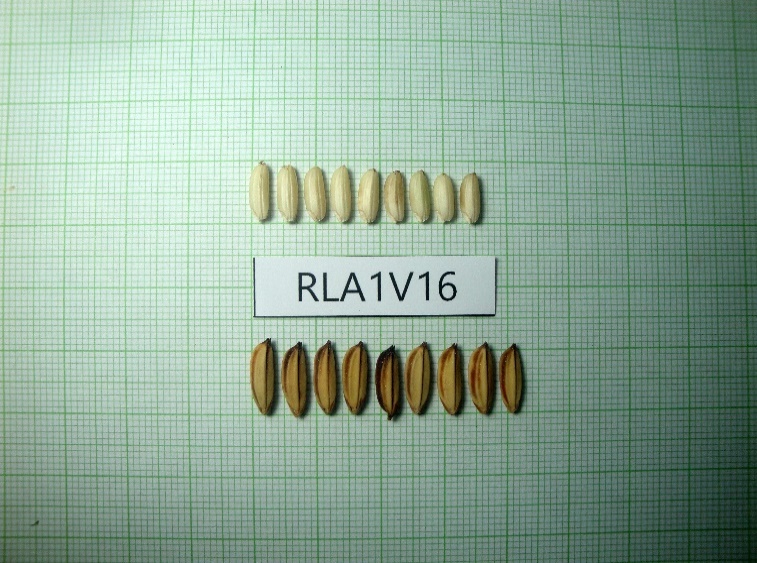** | |
| 90 | LU1 | RLA1V19 | ข้าวขาว | Khao Khao | Off-white | 7.853 | 3.282 | 2.442 | Medium | japonica | 57199 | 3730 | 0.0652 | **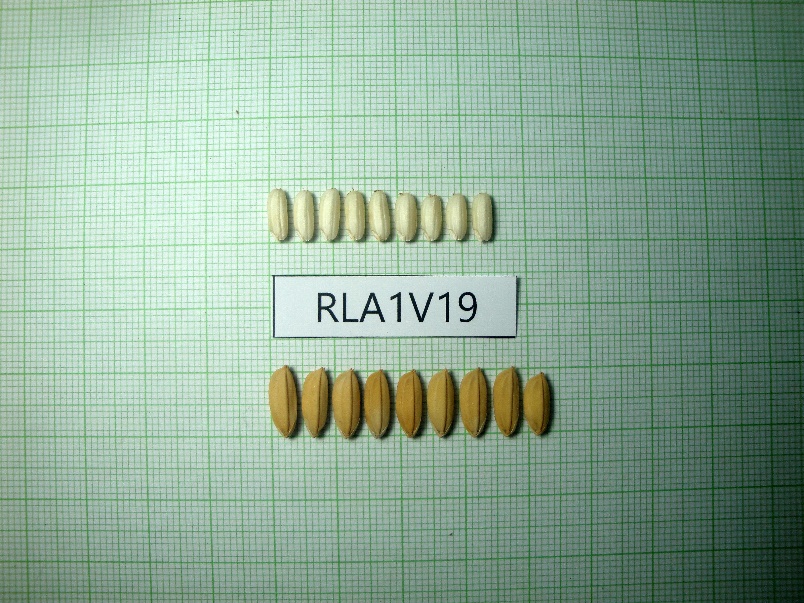** | |
| 91 | LU1 | RLA1V21 | ข้าวแดงบิด | Khao Daeng Bit | Off-white | 8.313 | 2.945 | 2.831 | Medium | japonica | 56763 | 2503 | 0.0441 | **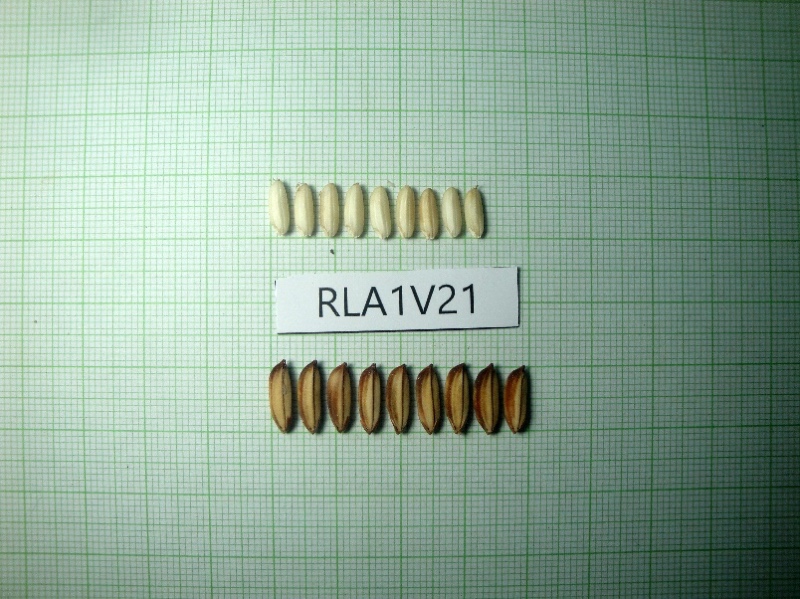** | |
| 92 | LU1 | RLA1V22 | ข้าวลืมผัว | Khao Luem Phua | Black | 8.072 | 2.945 | 2.686 | Medium | japonica | 29129 | 1746 | 0.0599 | **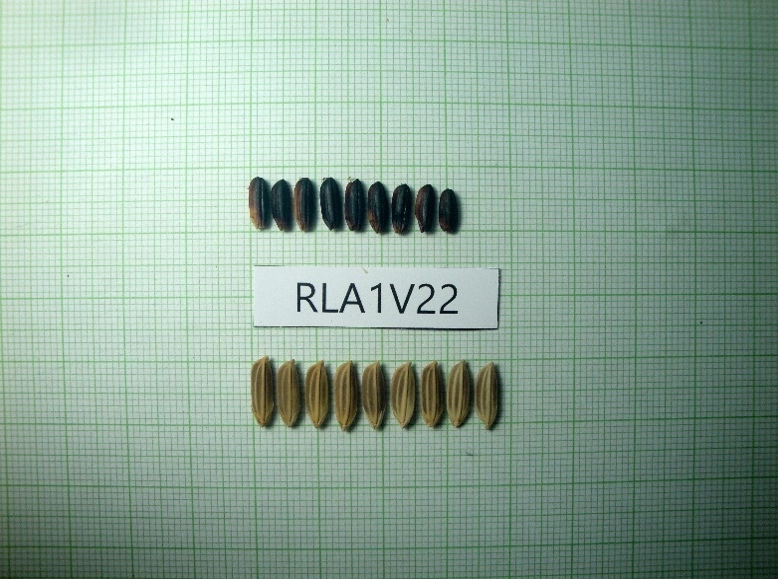** | |
| 93 | LU1 | RLA1V23 | ข้าวจ้าว | Khao Chao | Light | 8.798 | 2.501 | 3.581 | Slender | indica | 56088 | 9633 | 0.1717 | **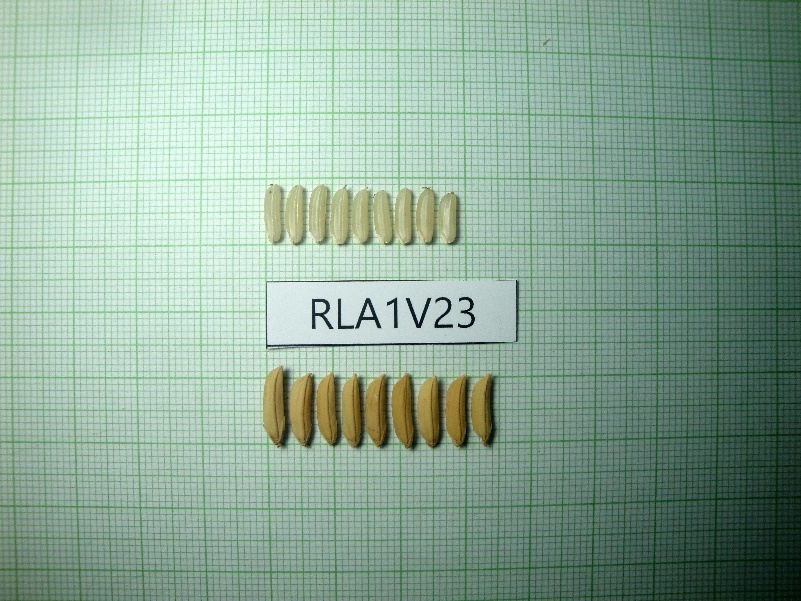** | |
| 94 | LU1 | RLA1V24 | ข้าวก่ำแดง | Khao Kam Daeng | Black | 8.193 | 3.056 | 2.677 | Medium | japonica | 33491 | 1898 | 0.0567 | **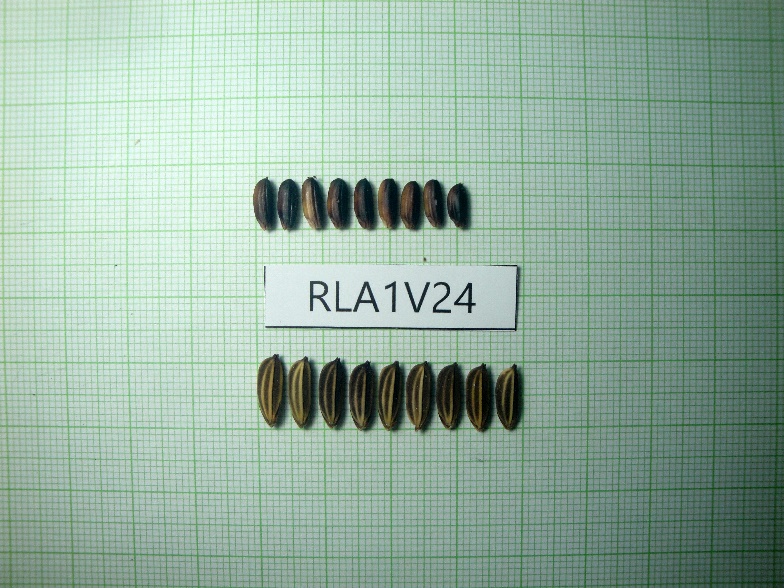** | |
| 95 | LU1 | RLA1V25 | ข้าวแดง+ข้าวเหลือง+ข้าวแพร่ | Khao Daeng + Khao Luang + Khao Phrae | Off-white | 8.675 | 3.278 | 2.619 | Medium | indica | 31663 | 15398 | 0.4863 | **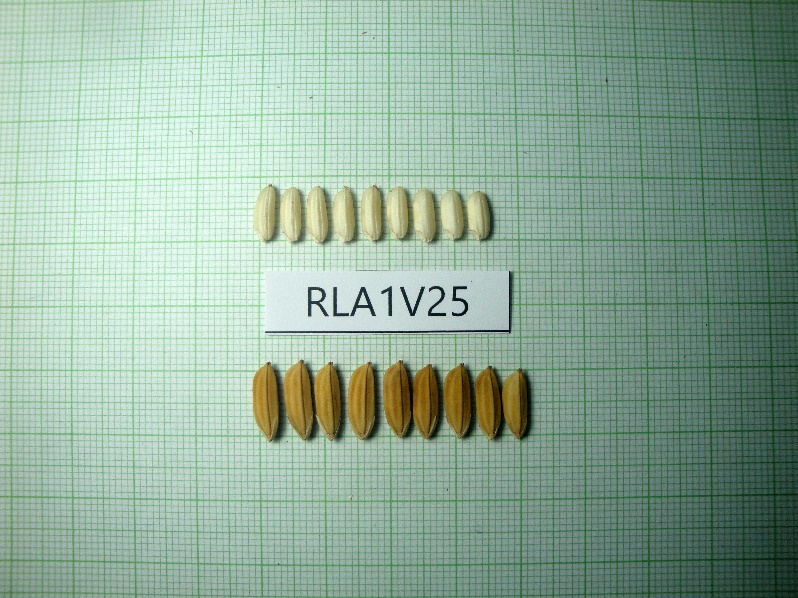** | |
| 96 | LU2 | RLA2V01 | ข้าวลาย | Khao Lai | Off-white | 7.992 | 3.021 | 2.724 | Medium | japonica | 30984 | 2117 | 0.0683 | **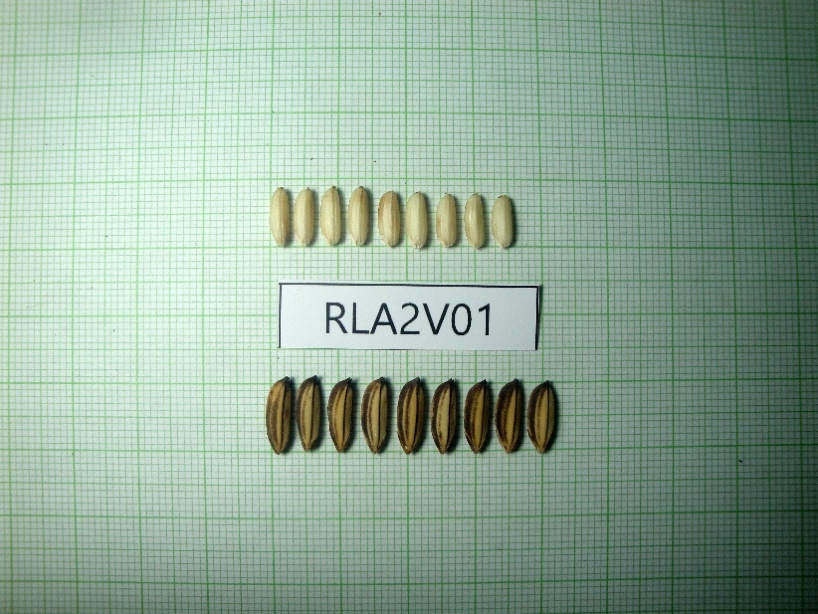** | |
| 97 | LU2 | RLA2V02 | ข้าวนอน | Khao Non | Off-white | 6.442 | 3.093 | 2.127 | Medium | japonica | 28762 | 1855 | 0.0645 | **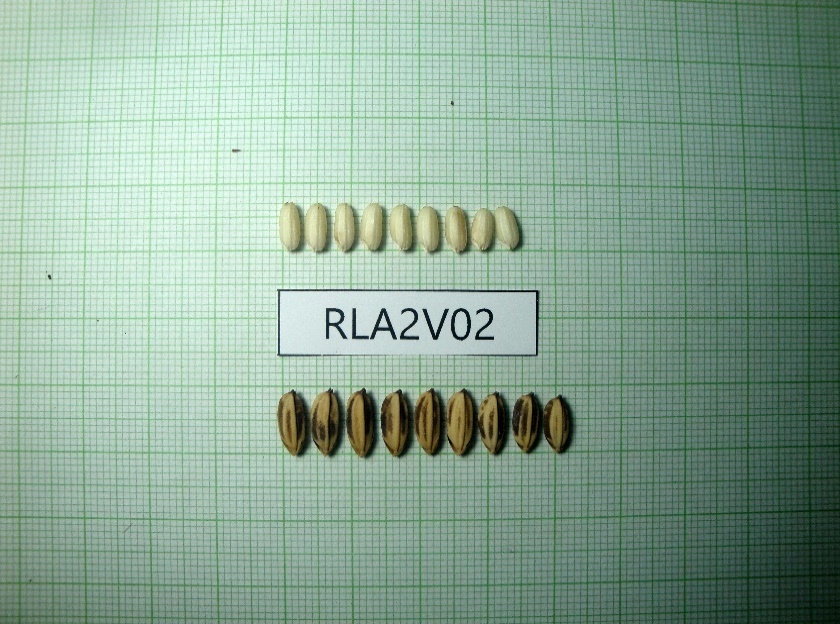** | |
| 98 | LU2 | RLA2V03 | ข้าวกลาง | Khao Klang | Off-white | 8.214 | 3.237 | 2.609 | Medium | japonica | 29160 | 1712 | 0.0587 | **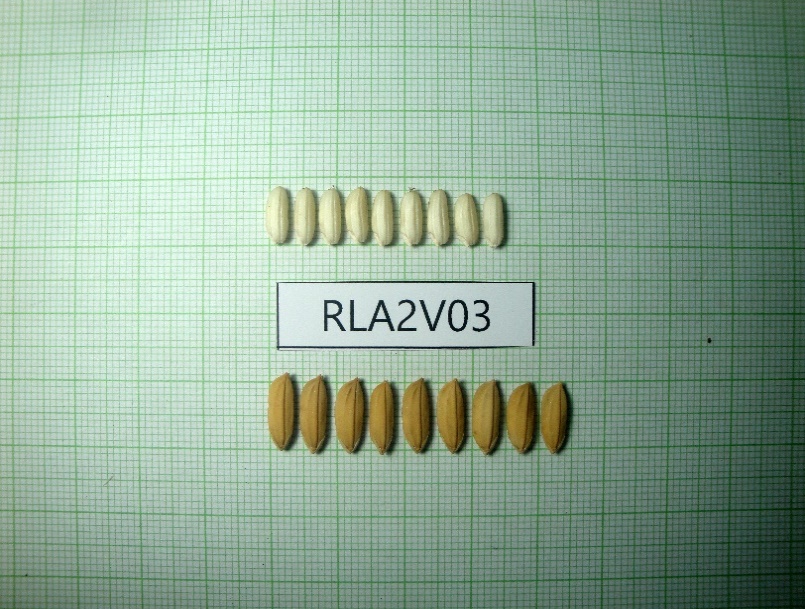** | |
| 99 | LU2 | RLA2V04 | ข้าวปีก | Khao Pik | Off-white | 7.329 | 3.094 | 2.355 | Medium | japonica | 32648 | 1566 | 0.048 | **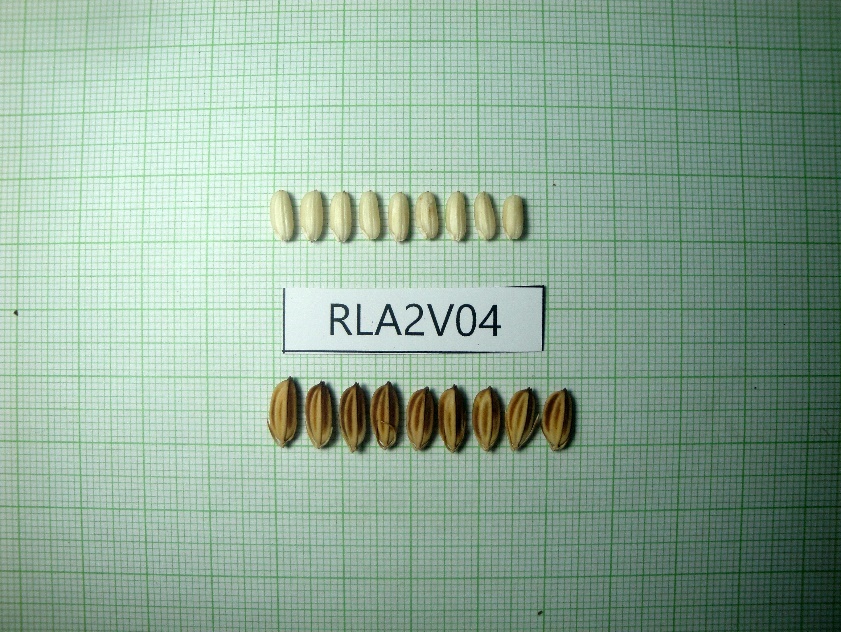** | |
| 100 | LU2 | RLA2V07 | ข้าวปีก | Khao Pik | Off-white | 6.937 | 2.985 | 2.309 | Medium | japonica | 31787 | 1542 | 0.0485 | **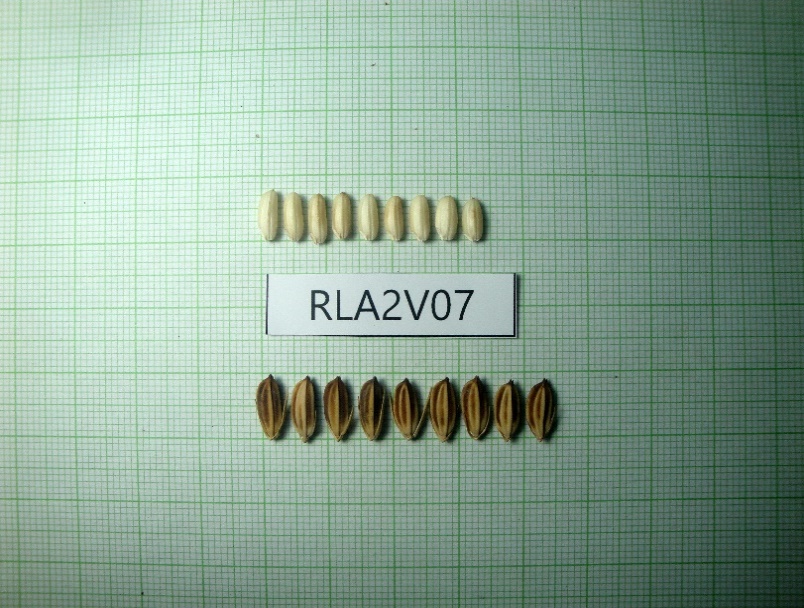** | |

**Supplementary Table 2. Fisher’s exact test of rice pericarp color (top) and seed shape (bottom) distribution comparing Khmuic and Palaungic ethnolinguistic groups.**

| **Group** | **Color** | **Japonica** | **p-value** | **Indica** | **p-value** |
| --- | --- | --- | --- | --- | --- |
| Khmuic | light | 1 | 0.0001** | 1 | 0.0023** |
|  | white | 0 |  | 1 |  |
|  | off_white | 32 |  | 2 |  |
|  | red | 0 |  | 0 |  |
|  | brown | 1 |  | 0 |  |
|  | black | 8 |  | 3 |  |
| Palaungic | light | 23 |  | 12 |  |
|  | white | 0 |  | 0 |  |
|  | off_white | 3 |  | 4 |  |
|  | red | 1 |  | 5 |  |
|  | brown | 0 |  | 0 |  |
|  | black | 4 |  | 0 |  |

**Significance at P<0.01

| **Group** | **Shape** | **Japonica** | **p-value** | **Indica** | **p-value** |
| --- | --- | --- | --- | --- | --- |
| Khamuic | Bold | 2 | 0.6062 | 0 | 1.0000 |
|  | Medium | 37 |  | 6 |  |
|  | Slender | 3 |  | 1 |  |
| Palaungic | Bold | 2 |  | 1 |  |
|  | Medium | 24 |  | 17 |  |
|  | Slender | 4 |  | 3 |  |

**Supplementary Table 3. Genetic diversity indices of 100 rice samples categorized by language branch and rice type**

| **Branch** | **Rice type** | **No. of individual** | **He** | **Ho** | **Ne** |
| --- | --- | --- | --- | --- | --- |
| Khmuic | Japonica | 42 | 0.0957 | 0.0589 | 1.1058 |
|  | Indica | 7 | 0.3439 | 0.3768 | 1.5242 |
| Palaungic | Japonica | 30 | 0.1057 | 0.0564 | 1.1182 |
|  | Indica | 21 | 0.3207 | 0.3129 | 1.4722 |

**Supplementary Table 4. Mantel and partial Mantel test results assessing the influence of linguistic, geographic, and elevational factors on genetic differentiation in the Japonica and Indica rice groups**

| **Group** | **Factor (control)** | **Mantel_r** | **P_value** |
| --- | --- | --- | --- |
| **Japonica** | Language | 0.126479523709381 | 0.001** |
|  | Geography | 0.0914722342834942 | 0.036* |
|  | Elevation | -0.0919760806290396 | 0.937 |
|  | Language (Geography) | 0.0989243696584717 | 0.034* |
|  | Language (Elevation) | 0.155088214148418 | 0.001** |
|  | Geography (Language) | -0.0459126410899883 | 0.731 |
|  | Geography (Elevation) | 0.140694274966337 | 0.001** |
|  | Elevation (Language) | -0.128750095927163 | 0.989 |
|  | Elevation (Geography) | -0.141018593094729 | 0.992 |
| **Indica** | Language | 0.0397426689407467 | 0.355 |
|  | Geography | 0.112325802830491 | 0.116 |
|  | Elevation | 0.244890871718331 | 0.016* |
|  | Language (Geography) | -0.0841335846772413 | 0.821 |
|  | Language (Elevation) | -0.0615681897833449 | 0.686 |
|  | Geography (Language) | 0.134369917462261 | 0.003** |
|  | Geography (Elevation) | -0.00880665113185349 | 0.515 |
|  | Elevation (Language) | 0.249105164008688 | 0.026* |
|  | Elevation (Geography) | 0.219165235607258 | 0.046* |

*Significance at P<0.05; **Significance at P<0.01
